# Genome sequencing of the Australian wild diploid species *Gossypium australe* highlights disease resistance and delayed gland morphogenesis

**DOI:** 10.1111/pbi.13249

**Published:** 2019-09-13

**Authors:** Yingfan Cai, Xiaoyan Cai, Qinglian Wang, Ping Wang, Yu Zhang, Chaowei Cai, Yanchao Xu, Kunbo Wang, Zhongli Zhou, Chenxiao Wang, Shuaipeng Geng, Bo Li, Qi Dong, Yuqing Hou, Heng Wang, Peng Ai, Zhen Liu, Feifei Yi, Minshan Sun, Guoyong An, Jieru Cheng, Yuanyuan Zhang, Qian Shi, Yuanhui Xie, Xinying Shi, Ying Chang, Feifei Huang, Yun Chen, Shimiao Hong, Lingyu Mi, Quan Sun, Lin Zhang, Baoliang Zhou, Renhai Peng, Xiao Zhang, Fang Liu

**Affiliations:** ^1^ State Key Laboratory of Cotton Biology, Henan Key Laboratory of Plant Stress Biology School of Life Sciences Bioinformatics Center School of Computer and Information Engineering Henan University Kaifeng China; ^2^ State Key Laboratory of Cotton Biology Institute of Cotton Research Chinese Academy of Agricultural Sciences Anyang China; ^3^ School of Life Science and Technology Henan Institute of Science and Technology Collaborative Innovation Center of Modern Biological Breeding of Henan Province Henan Key Laboratory Molecular Ecology and Germplasm Innovation of Cotton and Wheat Xinxiang China; ^4^ Guangzhou Genedenovo Biotechnology Co. Ltd Guangzhou China; ^5^ Anyang Institute of Technology Anyang China; ^6^ Nanjing Agricultural University Nanjing China

**Keywords:** *Gossypium australe*, genome sequencing, resistance, Verticillium wilt, delayed gland morphogenesis, gene function

## Abstract

The diploid wild cotton species *Gossypium australe* possesses excellent traits including resistance to disease and delayed gland morphogenesis, and has been successfully used for distant breeding programmes to incorporate disease resistance traits into domesticated cotton. Here, we sequenced the *G. australe* genome by integrating PacBio, Illumina short read, BioNano (DLS) and Hi‐C technologies, and acquired a high‐quality reference genome with a contig N50 of 1.83 Mb and a scaffold N50 of 143.60 Mb. We found that 73.5% of the *G. australe* genome is composed of various repeat sequences, differing from those of *G. arboreum* (85.39%), *G. hirsutum* (69.86%) and *G. barbadense* (69.83%). The *G. australe* genome showed closer collinear relationships with the genome of *G. arboreum* than *G. raimondii* and has undergone less extensive genome reorganization than the *G. arboreum* genome. Selection signature and transcriptomics analyses implicated multiple genes in disease resistance responses, including *GauCCD7* and *GauCBP1*, and experiments revealed induction of both genes by *Verticillium dahliae* and by the plant hormones strigolactone (GR24), salicylic acid (SA) and methyl jasmonate (MeJA). Experiments using a Verticillium‐resistant domesticated *G. barbadense* cultivar confirmed that knockdown of the homologues of these genes caused a significant reduction in resistance against *Verticillium dahliae*. Moreover, knockdown of a newly identified gland‐associated gene *GauGRAS1* caused a glandless phenotype in partial tissues using *G. australe*. The *G. australe* genome represents a valuable resource for cotton research and distant relative breeding as well as for understanding the evolutionary history of crop genomes.

## Introduction

In modern agricultural ecosystem, the narrow genetic base of modern crop cultivars, in which diversity has been lost in domestication, is becoming a major bottleneck for crop improvement programmes, especially for cultivated cotton. The use of close relatives of domesticated plants or crop wild relatives (CWRs) is a promising approach to enhance the genetic diversity and resistance to biotic and abiotic stresses of cultivated crops (Mammadov *et al*., 2018). Genomic analyses of CWRs generate data that support the use of CWRs to expand the genetic diversity of crop plants, which will strongly promote biodiversity, agricultural sustainability and food security (Brozynska *et al*., 2016). It is becoming increasingly important for genomic studies on CWRs; many such reports published in 2018 and 2019 (Arora *et al*., 2019; Milner *et al*., 2019), including wild rice (Zhao *et al*., 2018), wild wheat (Thind *et al*., 2018), soya bean wild relatives (Xie *et al*., 2019), wild tomato (Schmidt *et al*., 2017), wild peanut (Yin *et al*., 2018) and so on.

The *Gossypium* genus is highly diverse and includes the highest economically valuable species among all field crops. This genus is of great significance for plant research studies of plant taxonomy, polyploidization, phylogeny, cytogenetics and genomics (Kunbo and Jonathan, 2018). The wealth of diversity available among wild cotton species is a valuable resource for cotton breeders. There are diverse *Gossypium* taxa across the world: the A, B, E and F *Gossypium* genomes are distributed in Asia and Africa, the C, G and K genomes are found in Australia, and the D and AD genomes are distributed in the Americas and Pacific islands (Wendel *et al*., 2010). To date, cotton genomics researchers have developed high‐quality reference sequences for 2 diploid groups, as well as allotetraploid cottons (‘A‐genome’, ‘D‐genome’ and ‘AD‐genome’ clade) including 3 cultivated species (AA, *G. arboreum*, AADD, *G. hirsutum* and *G. barbadense*) and 1 wild ancestor species (DD, *G. raimondii*) (Du *et al*., 2018; Hu *et al*., 2019; Li *et al*., 2014, 2015; Paterson *et al*., 2012; Wang *et al*., 2012, 2019a; Zhang *et al*., 2015). Comparatively little research attention has been focused on Australian cotton species such as *G. australe* (with its ‘GG’ genome in cotton genomics nomenclature) (Chen *et al*., 2014; Liu *et al*., 2015; Wang *et al*., 2018c).

*Gossypium* species are characterized by their lysigenous glands containing terpenoids, important secondary phytoalexins consisting predominantly of the aldehyde gossypol, which constitute an important plant agent against pests and diseases in cotton (Bell, 1969; Cai *et al*., 2010; Gao *et al*., 2013; Tian *et al*., 2018). However, gossypol deposited in the glands of *Gossypium* is toxic to nonruminant animals and humans, while glandless cotton varieties (*i.e*. glandless in both seeds and plants) have no or very low gossypol content, with their resistance to pests and disease being attendantly reduced (Cherry, 1983; Mcmichael, 1959; Vaissayre and Hau, 1985).

*Gossypium australe* has been an important resource in the era of modern cotton genomics. Specifically, *G. australe* is highly resistant to Verticillium wilt disease (Benkang and Cun, 1996; Gu *et al*., 1993; Wang *et al*., 2018a) and is therefore viewed and has already been used as an important germplasm resource for the genetic improvement of cultivated upland cotton to increase resistance to *Verticillium dahliae,* seeking the inducement and identification of chromosome introgressions and translocations into *G. hirsutum*, as well as in the construction of a complete set of alien chromosome addition lines into *G. hirsutum* that were generated with aim of exploiting *G. australe*'s distinct traits including glanded‐plant and glandless‐seed and resistances to pests and diseases (Benbouza *et al*., 2009; Chen *et al*., 2014; Liu *et al*., 1999; Wang *et al*., 2018c).

As with other crops, especially polyploid crops, there have been a variety of cotton improvement strategies based on utilization of natural germplasm resources of wild relative and progenitor species to improve plant resistance to diseases such as Verticillium and Fusarium wilt, as well as to pests and abiotic stresses like drought. For example, the Australian wild species *Gossypium australe*—which exhibits delayed gland morphogenesis wherein the dormant seeds are glandless (low gossypol content) but the germinated cotyledons are glanded (Benkang and Cun, 1996; Gu *et al*., 1993; Wang *et al*., 2018a; Wendel *et al*., 1991)—has been used to generate glandless‐seed but glanded‐plant commercial cotton varieties, thereby providing seeds that lack gossypol and are suitable for food and feed uses but also with strong plant resistance to cotton pests and diseases (Ma *et al*., 2016; Wendel *et al*., 1991).

Here, to better enable the continued use of this distinctive cotton wild relative species with its prominent traits like resistance to disease and delayed gland morphogenesis, we performed *de novo* sequencing of *G. australe* genome and focused on disease resistance and the *G. australe*‐specific gland development traits. We used an integrated approach combining four separate sequencing technologies to assemble a high‐quality reference genome sequence. Through genome sequencing of *G. australe* and transcriptome analysis, we explored the evolution and adaptability of Australian *Gossypium* species and investigated disease resistance genes and gland formation genes. We hope to provide a theoretical and applied basis for the discovery of the molecular mechanisms underlying the beneficial characteristics of this species and promote cotton breeding for the sustainable development of agriculture, benefiting food security despite the threats of biotic and abiotic stresses.

## Results

### Genome sequencing and construction of a high‐quality genome assembly for *G. australe*

**Figure 1:**
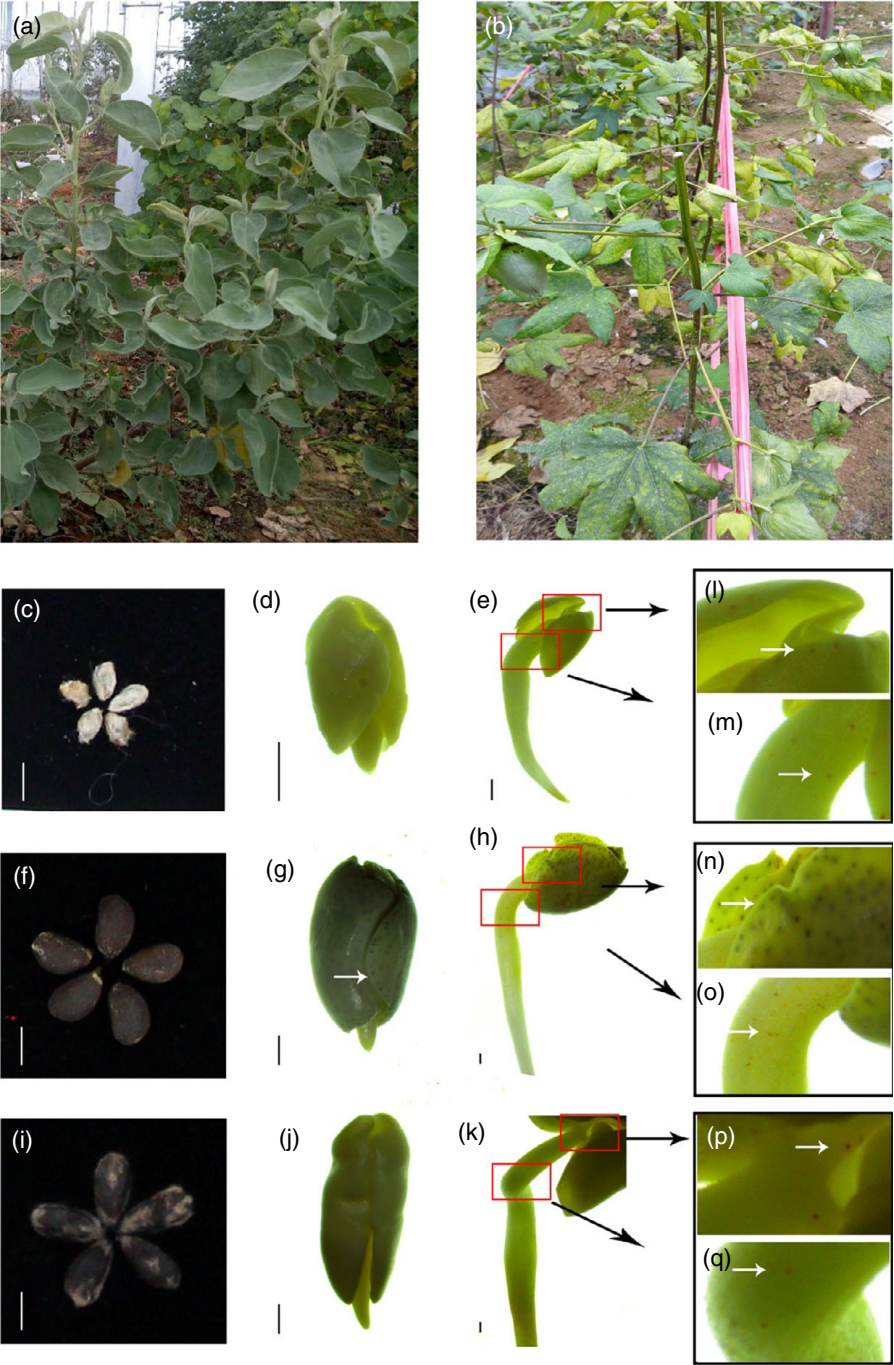
The plants of *G. australe* and *G. arboretum*, and the forming of new glands during seed germination of *G. australe*,* G. arboretum*,* G. hirsutum* (Xiangmian 18). (a) *G. australe* plant, resistant or immune to Verticillium wilt. (b) *G. arboretum* plant, susceptible to Verticillium wilt. (c, f and i) are the delinted seeds of *Gossypium australe*,* G. arboreum* and *G. hirsutum*, respectively. Scale bar, 5 mm. (d and e) are two germination stages of *G. australe*, early stage before GF (gland formation), beginning stage of GF; (g and h) are the same two germination stages of *G. arboreum*. (J and k) are stages of Xiangmian 18 (*G. hirsutum*), scale bars, 1 mm. (l and m) are enlarged versions of the positions indicated by the red box in Figure (e). (n and o) correspond to (h), and (p and q) correspond to (k). The white arrow indicates the location of the glands.

*Gossypium australe* has shown excellent resistance to the fungus disease Verticillium wilt, and the disease had little influence on morphology of *G. australe* plants (Figure 1a,b); in contrast, the stems and leaves of *G. arboreum* cultivar plants were greatly damaged after infection with Verticillium wilt. Another prominent trait of *G. australe* is delayed gland morphogenesis, and the seeds of *G. australe* (Figure 1c,d,e) have no gland in seed, but were observed during seeds germinate process, differing from that in *G. arboretum,* which is glanded in whole plants (including seeds) (Figure 1f,g,h). This precious resource could facilitate glandless‐seed and glanded‐plant cotton breeding and provide seeds lacking gossypol for food or feed, as well as maintain the resistance of cotton to pests and diseases.

**Figure 2:**
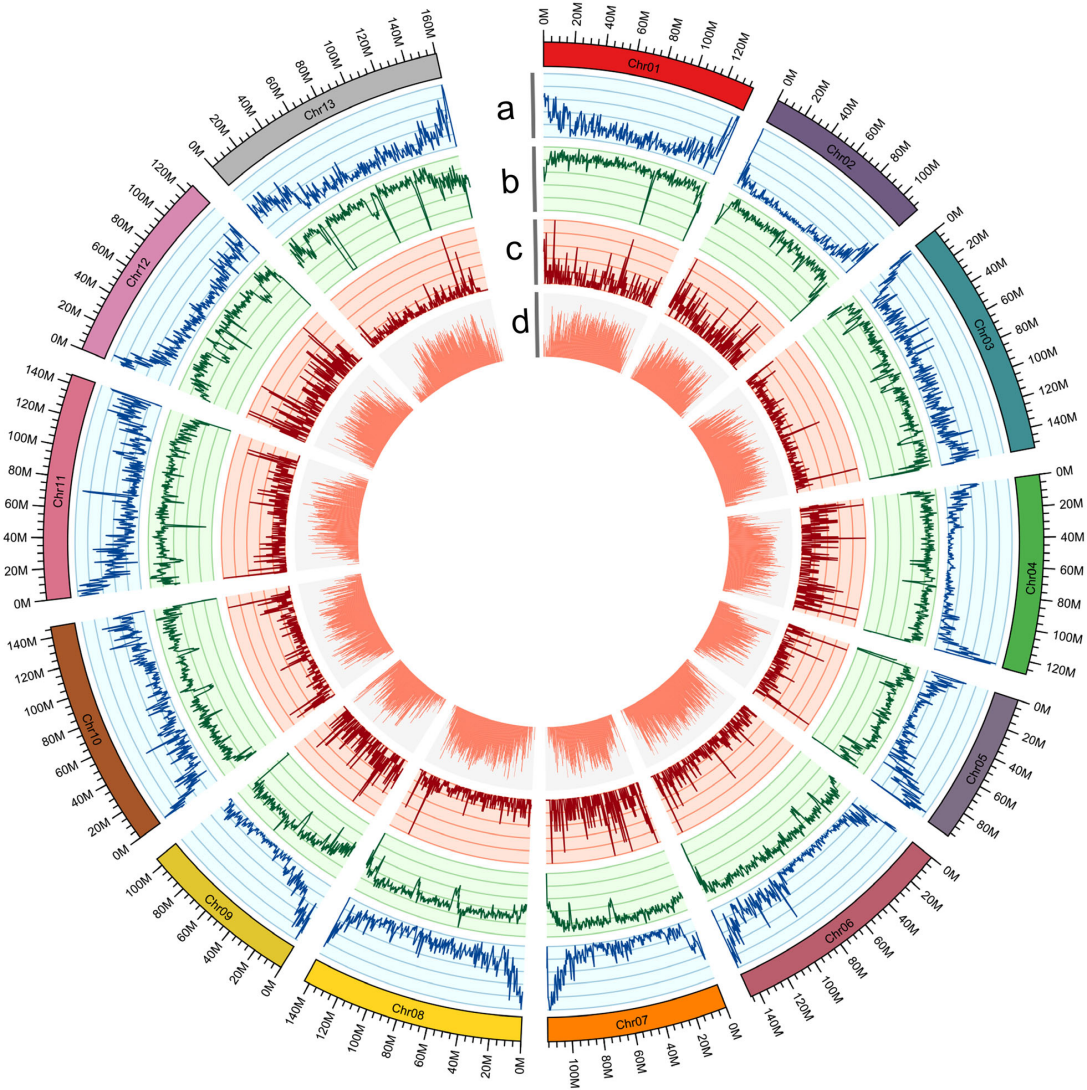
Characterization of the *G. australe* cotton genome. (a) Gene density in each chromosome; (b) transposable element (TE) density in each chromosome; (c) ncRNA density in each chromosome; (d) GC content in each chromosome.

**Figure 3:**
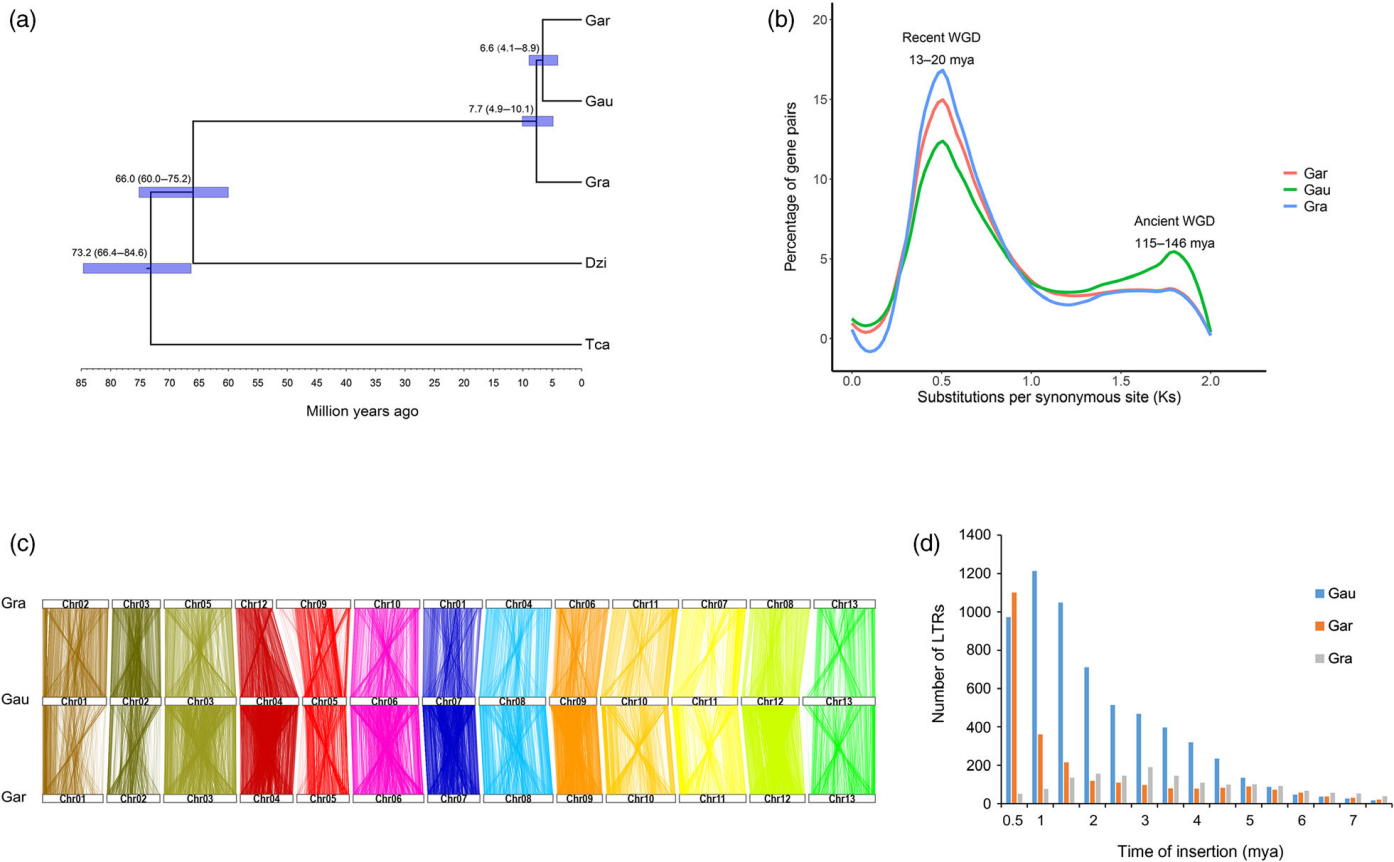
Phylogenetic and evolutionary analysis of the *Gossypium* genomes. (a) Phylogenetic analysis indicated that *G. australe* and *G. arboreum* diverged 6.6 (4.1–8.9) million years ago (mya). Gra: *G. raimondii*. Gar: *G. arboreum*; Gau: *G. australe*; Dzi: *Durio zibethinus*; Tca, *Theobroma cacao*. (b) Ks analyses suggested that the *Gossypium* genomes might have undergone two WGD events. (c) Many collinear blocks were found when comparing either the *G. raimondii* (Gra) or *G. arboreum* (Gar) genome with the *G. australe* (Gau) genome. Numbered rectangles represent the chromosomes. (d) Analysis of the LTR number and insertion time in *G. australe* (Gau), *G. arboreum* (Gar) and *G. raimondii* (Gra).

Thus, we sequenced and assembled the *G. australe* genome with a combination of four technologies: Pacbio single‐molecule real‐time (SMRT) sequencing, paired‐end sequencing, optical mapping (DLS) and Illumina short‐read Hi‐C. Assembly with these complementary data types proceeded in a stepwise fashion, producing progressively improved assemblies (Table 1, Table S1). The initial assembly of the single‐molecule real‐time sequencing data alone resulted in a contig N50 (the minimum length of contigs accounting for half of the haploid genome size) of 2.50 Mb. PacBio contigs were first scaffolded using large‐insert pair‐end library reads, which resulted in a scaffold N50 of 3.59 Mb. The sequences were then scaffolded and corrected using optical mapping data (Figure S1), and the resulting scaffolds were clustered into chromosome‐scale scaffolds using Hi‐C data (FigureS2). With K‐mer distribution analysis, the genome size was estimated to be 1.67 Gb (FigureS3), which is very similar to an earlier prediction (Hendrix and Stewart, 2005). The final assembly comprised 1.75 Gb of sequence with a contig N50 *of* 1.83 Mb and a scaffold N50 of 143.60 Mb, with only 650 scaffolds covering the 13 haploid *G. australe* chromosomes (Figure 2, Tables 1, S1).

**Table 1:** Summary of genome assembly and annotation for *G. australe*

Genomic feature	*G. australe*
Total length of contigs (bp)	1 729 091 355
Total length of assemblies (bp)	1 752 741 698
Estimated gap size (bp)	23 650 343
Percentage of anchoring	99.08%
Percentage of anchoring and ordering	98.99%
Number of contigs	2598
Contig N50 (bp)	1 825 353
Contig N90 (bp)	453 340
Number of scaffolds	650
Scaffold N50 (bp)	143 600 552
Scaffold N90 (bp)	104 992 986
GC content	36.39%
Percentage of repeat sequences	73.50%
Number of genes	40,694
Number of transcripts	45 350

We used the BUSCO method based on a benchmark of 1440 highly conserved core plant genes to further evaluate assembly quality and completeness (Kriventseva *et al*., 2019), which revealed that 95.9% of the genes were present in our assembly and indicating that this *G. australe*, genome assembly is nearly complete (Table S5). Further, the accuracy of the assembly was supported by alignment of the Illumina short‐read data, resulting in a 97.48% mapping ratio. The genome assembly completeness was also validated by aligning full‐length transcripts derived from SMRT sequencing to the assemble genome, which a total of 99% of the 158,566 full‐length transcripts from the *G. australe* ovules and leaves were detected in our assembly (Table S2).

### Annotation of the *G. australe* genome

**Figure 4:**
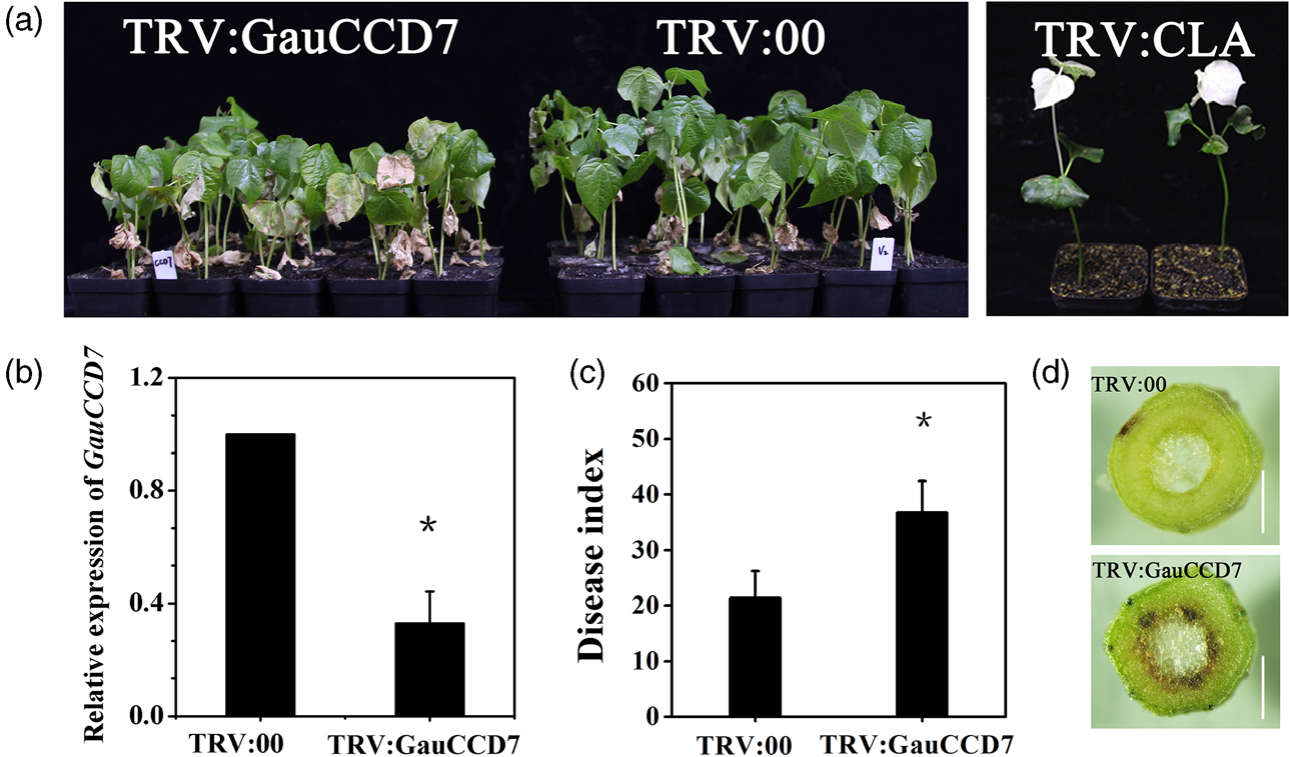
*GauCCD7* positively regulates cotton defence against *V. dahliae* in Xinhai 15. (a) Disease symptoms of TRV:GauCCD7 (left) and TRV:00 plants (centre) inoculated with *V. dahliae* strain V991, which were photographed 15 days after inoculation, and the albino phenotype of the plants inoculated with TRV:CLA after 15 days (right). (b) (qRT‐PCR) Analysis of the expression of *GauCCD7* in TRV:00 and TRV:GauCCD7. Statistical analyses were performed using Student's *t* test: *P < 0.05. (c) The disease index and incidence rate in TRV:00 and TRV:GauCCD7 were measured at 17 dpi (days postinoculation). Three biological replicates with at least 35 plants per replication. (d) Section anatomy in the stem was observed 17 days after *V. dahliae* treatment of TRV:00 and TRV:GauCCD7. Bars, 1 mm.

We annotated 40 694 gene models in the *G. australe* genome by combining *ab initio* gene prediction, homolog protein data searches and the sequences of the aforementioned full‐length transcripts (Figure 2a), and a number similar to the 40 976 and 40 960 consensus protein‐coding‐gene models previously predicted for the *G. raimondii* and *G. arboreum* genomes, respectively (Du *et al*., 2018; Wang *et al*., 2012). Approximately 97% of the predicted *G. australe* gene models were annotated by BLAST in four databases, including Nr, Swiss‐prot, KOG and KEGG (Table S3). Additionally, the *G. australe* genome is predicted to encode 1366 rRNAs, 1292 tRNAs, 339 microRNAs (miRNAs) and 3388 small nuclear RNAs (snRNAs) (S4). Orthologous clustering of the *G. australe* predicted proteome with 7 closely related plant genomes identified 95 666 gene families in common, with 15 696 gene families that were present specifically in *G. australe* (FigureS4).

Notably, 73.5% of the *G. australe* genome is composed of various types of repeat sequences (Figure 2b), a proportion distinct from the reported repeat sequence content of *G. arboreum* (85.39%), *G. hirsutum* (69.86%) and *G. barbadense* (69.83%) (Du *et al*., 2018; Wang *et al*., 2019a), and long terminal repeat (LTR) retrotransposons accounted for 92.9% of these sequences in the *G. australe* genome (Table S6).

Compared with those in the *G. raimondii* genomes, Gypsy elements showed noticeable proliferation in both the *G. australe* and *G. arboreum* genomes, whereas Copia elements have apparently preferentially accumulated in the *G. raimondii* genome (Table S7). Our results are consistent with previous reports that the detected differences in transposable elements (TEs) between the *G. raimondii* genome and the other two cotton genomes were established during the divergence of *G. raimondii* and the common ancestor of *G. australe* and *G. arboreum* before the divergence of these two genomes (Hawkins *et al*., 2006).

Furthermore, a similar proportion of Gypsy subgroup LTR (Llorens *et al*., 2009) was observed between the *G. australe* and *G. arboreum* genomes, with the CRM subgroup being predominant among the Gypsy elements of both genomes. However, a significantly lower proportion of the CRM subgroup than those of *G. australe* and *G. arboretum* and different proportions of other subgroups from *G. australe* and *G. arboreum* were observed in *G. raimondii*, which was consistent with the predominance of Copia in the retrotransposons of *G. raimondii* (Table S8).

### Genome evolution in *G. australe*

The *Gossypium* genus comprises 8 diploid genome groups (A through G and K), as well as one allopolyploid clade (AD genome) formed from an ancient merger and chromosome doubling from A and D genome ancestors (Kunbo and Jonathan, 2018). Through molecular phylogenetic analyses, we showed that a divergence time for *G. australe* and *G. arboreum* of 6.6 (4.1–8.9) million years ago, and their common ancestor had diverged from *G. raimondii of* 7.7 (4.9–10.1) million years ago (Figure 3a) (Carvalho *et al*., 2011).

The *G. australe* genome was scanned for syntenic gene blocks. We calculated the age distribution for all duplicate gene pairs based on the substitution per synonymous site (Ks) values. We detected a large peak centred around Ks values of approximately 0.5 for *G. australe*, very similar to the peak detected in the two previously reported diploid cotton genomes (Figure 3b); this apparent whole‐genome duplication (WGD) event has been previously estimated to have occurred 13–20 million years ago (Li *et al*., 2014; Wang *et al*., 2012). Our results thus further support that this recent whole‐genome duplication (WGD) event occurred in all of the cotton genomes based on the phylogenetic tree of the *Gossypium* genus (Hawkins *et al*., 2006). Notably, a whole‐genome alignment approach revealed that the *G. australe* genome showed closer collinear relationships with the genome of *G. arboreum* than of *G. raimondii*, a finding consistent with multiple reported phylogenetic analyses. Collinear blocks covered 72% of the *G. arboreum* genome and 71% of the *G. australe* genome, but covered only 60% of the *G. raimondii* genome and 60% of the *G. australe* genome (Figure 3c, Figure S5).

Extensive evidence in plant genomics supports the understanding that the expansion of TE families is one of the major factors that influence genome evolution. Our LTR analysis indicated that the retrotransposition activity of *G. australe* apparently increased continuously from 7.5 million years ago until about 1 million years ago, before subsequently decreasing (Figure 3d). Notably, with the exception of the most recent 0.5 million years, *G. australe* apparently had higher retrotransposition activity than did *G. arboreum,* a situation consistent with the different genome sizes of these three genomes. However, *G. australe* harbours more than twofold more intact LTRs than *G. arboreum*, with a similarly sized genome and similar TE ratio between these two genomes, findings which strongly suggest that *G. australe* has experienced less genome reorganization than the domesticated *G. arboreum,* which we know has undergone several rounds of artificial selection.

### Gene evolution in *G. australe*

Positive selection plays important roles in plant evolution and adaptation to biotic and abiotic stresses; gene expression and regulation changes have been postulated to be key determinants of the rates of adaptive evolution (Khan *et al*., 2016; Paape *et al*., 2018; Seeholzer *et al*., 2010; Sun *et al*., 2018a; Zambounis *et al*., 2016). The positively selected genes (PSGs) between *G. australe* and *G. arboretum* remain unknown.

Here, we analysed the PSGs in *G. australe* to assess how it has adapted to diverse wild environments and to search for loci relating to its strong resistance to Verticillium wilt, a trait for which it is much more resistant than the domesticated *G. arboreum*. The analysis showed identified selection signatures at 670 and 232 PSGs in the *G. australe* and *G. arboretum* genomes, respectively (Figure S6, Data S1). Subsequent KEGG pathway analysis indicated that statistically significant (*P *< 0.05) enrichment among the *G. australe* PSGs for the ‘Other types of O‐glycan biosynthesis’ (ko00514) pathway (Figure S7), which was associated with cotton fibre strength in studies of in *G. arboreum* (Hernandez‐Gomez *et al*., 2017; Natalio *et al*., 2017). There was also significant enrichment among the *G. australe* PSGs for ‘Riboflavin metabolism’ (ko00740) (Asai *et al*., 2010; Boubakri *et al*., 2016; Deng *et al*., 2011) and GPI (Shen *et al*., 2017) (Figure S7), pathways which have been implicated in plant responses to biotic stresses.

Interestingly, analysis of the PSGs associated with the 12 enriched pathways of (Figure S7, Data S1) identified three genes from the carotenoid biosynthesis pathway, suggesting that these compounds or their downstream metabolites may be involved in *G. australe*'s resistance to fungal pathogens. Carotenoid cleavage dioxygenase 7 (CCD7) is involved in strigolactone biosynthesis by cleaving asymmetrically the 9–10 double bond in various linear and cyclic carotenoids (including branch‐inhibiting hormones and share symbiotic fungi) and showed an elevated Ka/Ks value (Figure S7). Previous reports have shown that CCD7 is involved in disease‐related mechanisms (Decker *et al*., 2017). Thus, we cloned the *GauCCD7* gene, which shared 97.97% identity in amino acid sequence with *G. barbadense*, and investigated its expression and potential function in disease resistance.

### Functional analysis of *G. australe* genes associated with resistance to Verticillium wilt

The enzyme carotenoid cleavage dioxygenase 7 (CCD7) is involved in strigolactone biosynthesis: it asymmetrically cleaves the 9‐10 double bond in various linear and cyclic carotenoids (including branch‐inhibiting hormones and stimulatory molecules for symbiotic fungi). Given that the *G. australe* locus encoding CCD7 exhibited an elevated Ka/Ks value (Figure S7), and considering that previous studies have reported a disease‐related function for CCD7 in plants (Decker *et al*., 2017), we investigated its expression and potential function in disease resistance in *G. australe*. We conducted a qPCR‐based analysis of *GauCCD7* expression in *G. australe, G. arboretum* and *G. raimondii*. qPCR analysis of roots, stems and leaves showed that the expression pattern of *GauCCD7* in *G. australe* was different from that in *G. arboreum* and *G. raimondii* (Figure S8). Experiments that applied a variety of plant hormones or *V. dahliae* Kleb isolate to 3‐week‐old *G. australe* plants revealed that *GauCCD7* expression was significantly induced by *V. dahliae* Kleb and by the plant hormones strigolactone (GR24), salicylic acid (SA) and methyl jasmonate (MeJA), but not by ethylene (applied as ethephon) or abscisic acid (ABA) (FiguresS9 and S10).

We also performed virus‐induced gene silencing (VIGS) experiments to examine the disease resistance‐related phenotypic changes resulting from the knockdown of the *GauCCD7* homologue in the Verticillium wilt resistant *G. barbadense* cultivar Xinhai 15. We first confirmed the ability of a silencing construct targeting *GauCCD7* to knockdown the expression of *GbCCD7* in Xinhai 15 upon infiltration of tobacco rattle virus (TRV) into the cotyledons of newly germinated seedlings (Figure 4b). Subsequently, 14 days infiltrated control and *GbCCD7*‐knockdown plants were inoculated with *V. dahlia* and the disease symptoms were monitored. Compared to controls, the disease index values were significantly increased in *GbCCD7*‐knockdown plants (Figure 4a,c), thereby experimentally implicating *GbCCD7* in *G. barbadense* defence responses to the fungal pathogen. Upon dissection of these plant materials, we also noted that that the *GbCCD7*‐knockdown plants exhibited a pronounced vascular browning phenotype (Figure 4d).

In addition, we conducted an RNA‐seq‐based transcriptomic analysis seeking differentially expressed genes associated with *G. australe*'s resistance responses to Verticillium wilt. Ultimately, we focused on the genes which were simultaneously (1) significantly up‐regulated in *G. australe* and (2) significantly down‐regulated in *G. arboreum* during infection with Verticillium wilt, criteria which identified 31 genes, including GAUG00007269 (*bHLH19‐like* isoform) and GAUG00028019 (calmodulin‐binding protein, CBP) (Figures S11 and S12, Table S9). Among them, GAUG00028019 was selected as a candidate resistance gene of interest because this gene shared 91.86% identity in amino acid sequence with that in *G. barbadense* (Table S9). Other cotton calmodulin‐binding proteins that was functionally associated with responses to biotic and abiotic stress have been reported (Qin *et al*., 2018; Sun *et al*., 2018b; Zheng *et al*., 2015). We investigated the expression of *GauCBP1* in *G. australe* comparing with *G. arboretum* and *G. raimondii* (Figure S13). qPCR analysis of root, stem and leaves showed that *GauCBP1* expression was induced by *V. dahliae* Kleb and by the plant hormones GR24, SA and MeJA, but not by ethylene or ABA (Figures S14 and S15).

We also explored the potential disease‐related functions of the *GauCBP1* homologue in the aforementioned Verticillium wilt‐resistant *G. barbadense* cultivar Xinhai 15. Having confirmed that the silencing construct does indeed knockdown expression of *GbCBP1* (Figure S16b), experiments similar to the aforementioned VIGS analysis of *GbCCD7* again showed that knockdown of this candidate resistance gene homologue resulted in a significant reduction in Xinhai 15's resistance against Verticillium wilt.

### Genes involved in gland formation and function

To explore mechanisms relating to *G. australe*'s delayed gland morphogenesis relative to other cottons, we analysed the embryo and leaf transcriptomes of six cottons, including three Australian diploid G subgroup wild cotton species *(G. australe*,* G. bickii* and *G. nelsonii*) and two different *G. hirsutum* cultivars (the glandless ZHONG12 and Xiangmian 18) (Figure 1j,k) with few gland in seed but glanded plant. Based on the results of our previous studies, in which 24 differentially expressed cDNAs were identified in the new gland‐forming stage of Xiangmian 18 through suppression subtractive hybridization (SSH) analyses (Cai *et al*., 2003, 2010), among them we identified a transcription factor GRAS (Belong to GRAS family, GAI, RGA, SCR). GRAS proteins are an important family of plant‐specific proteins named after the first three members: GIBBERELLIC‐ACID INSENSITIVE (GAI), REPRESSOR of GAI (RGA) and SCARECROW (SCR). In this study of the *G. australe* genome, we cloned the GRAS gene in *G. australe* based on the GRAS gene cloned from Xiangmian 18 and named it *GauGRAS1 (*one of GRAS family), and then investigated its expression and function.

The expression levels of *GauGRAS1* and *GauPGF*, a positive regulator of gland formation (Ma *et al*., 2016), were analysed in embryos of *G. australe*, glanded *G. hirsutum* (C5, Jinxianduanguozhi), dominant glandless Zhongmiansuo 12 and recessive glandless Zhongmiansuo 12. The results showed that both *GauGRAS1* and *GauPGF* were highly expressed in glanded *G. hirsutum* but showed very low expression in *G. australe* and the two glandless lines (Figure S17). These results indicated that *GauGRAS1* is associated with gland formation. We also investigated the expression patterns of *GauGRAS1* in the embryos and leaf of *G. australe* and *G. bickii by* qRT‐PCR and RNA‐seq (Figures S18 and S19). The results showed that *GauGRAS1* has different expression patterns from *GauPGF*. The relative expression level of *GauGRAS1* was significantly lower in the embryos than in the leaves for both *G. australe* and *G. bickii*, and the expression level of *GauPGF* was significantly higher in the embryos than in the leave for both *G. australe* and *G. bickii* (Figure S18).

These findings were consistent with the results of the transcriptomic analyses of *G. australe, G. bickii and G. nelsonii* (Figure S19). In addition, during seed germination of three cotton species, both *GauGRAS1* and *GauPGF* are up‐regulated in the gland‐forming stage compared to early stages before gland formation. The relative expression level of the *GauGRAS1* gene showed somewhat differences compared to that of *GauPGF* by RT‐PCR (Figure S20, Figure 1). The results indicated that *GauGRAS1* was associated with gland formation, but its expression pattern was different from that of *GauPGF*. Thus, the function of *GauGRAS1* was further analysed using VIGS technology.

**Figure 5:**
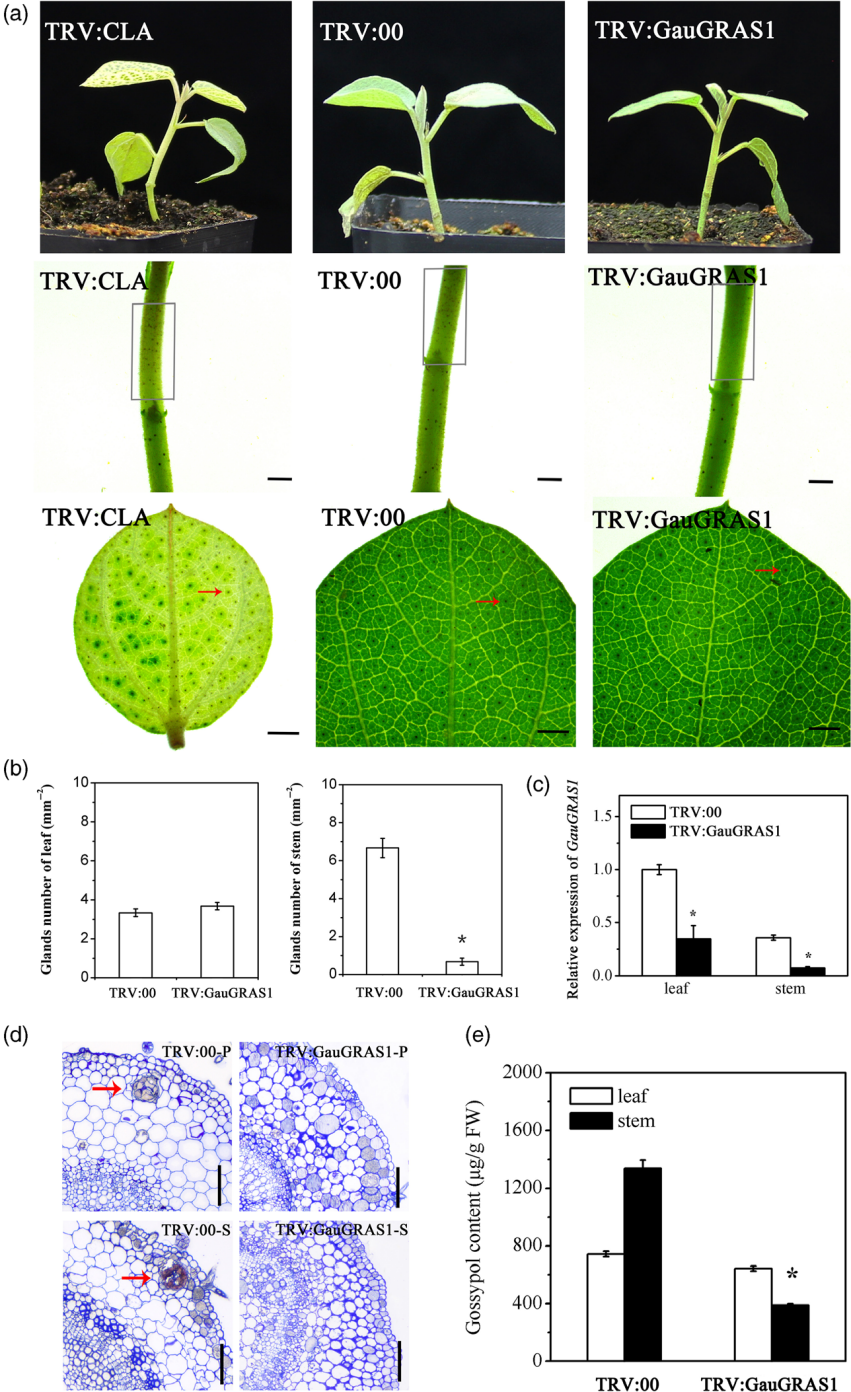
Functional characterization of *GauGRAS1* by VIGS. (a) Phenotypes of *Gossypium australe* after *GauGRAS1* silencing by VIGS; TRV:CLA and TRV:00 are the positive control and negative control, respectively. The grey box indicates glands in the stem, and the red arrow indicates glands on the leaf. Scale bars, 1 mm. (b) Statistical chart of the number of glands in the leaves and stems. (c) The silencing efficiency of *GauGRAS1*. (d) A cavity was observed in the empty vector (TRV:00) leaves and stems but disappeared in the *GauGRAS1*‐silenced plants. P: petiole, S: stem. Scale bars, 100 μm. (e) Gossypol content in empty vector (TRV:00) and in the *GauGRAS1*‐silenced leaves of *G. australe*. Error bars are the SD of three biological repeats. *P < 0.05; Student's *t* test, *n* = 3.

Suppressing *GauGRAS1* by VIGS led to glandless stems and petioles in *G. australe*, but the leaf glands did not change in *G. australe* (Figure 5a–c), and no glandular cavity was formed in the stems and petioles (Figure 5d). Glands still formed in true leaves, and the number of glands was not different from that in the control plant leaves (TRV:00) (Figure 5a,b). Moreover, the gossypol content in the stem of the *GauGRAS1*‐silenced plants was significantly reduced, but it remained almost unchanged in the leaves (Figure 5e). The functional results confirmed that the *GauGRAS1* gene was responsible for gland formation of partial tissues in *G. australe*, in contrast to *GauPGF*, which leads to glandlessness in all tissues, including the leaves and stems, in the *GauPGF*‐silenced plants of *G. australe* (Figure S21).

To better understand the evolution and function of gossypol/gland formation genes in the botanical system and *Gossypium*, we performed a comparative transcriptome analysis of the embryos and leaf using several glanded and glandless tetraploid cotton varieties. The differentially expressed genes (DEGs) were identified between glanded and glandless leaves, followed by module partition analysis based on weighted gene co‐expression network analysis (WGCNA). The magenta4 module was positively correlated with the presence/absence of glands (Figure S22, TableS10). Interestingly, the gene with the highest connectivity was glyoxalase I, which responds to stress in higher plants (Espartero *et al*., 1995; Hasanuzzaman *et al*., 2017), glutathione S‐transferase (Li *et al*., 2018) and Laccase 14 (Hu *et al*., 2018) that were probably associated with disease resistance (Table S10). In addition, 7 genes adjacent to *GoPGF* were co‐expressed with *GoPGF* in the magenta4 module (Table S11), indicating that function‐related genes are clustered together.

The pigment gland is a type of glandular trichome that can be found in approximately 30% of all vascular plant species (Huchelmann *et al*., 2017; Ma *et al*., 2016). To study the evolution history of gland formation genes, local collinearity was analysed based on forty genes adjacent to the *GoPGF* and *GRAS1* genes to assess the presence/absence in 30 sequenced genomes that represented several orders of plants (Figure S23). We found that the *GoPGF* gene evolved after the differentiation of dicotyledonous plants and monocotyledonous plants (Figure S24), and *GoPGF* did not originally function in glandular trichomes but later differentiated in *Gossypium* and other species. Furthermore, the *GRAS1* gene might be present earlier before the differentiation of the glandular trichome formation and regulate tissue‐specific genes’ expression. Later, GRAS1 acquired new regulatory functions for gland information in cotton (Figure S25).

The results suggested that the gland formation pathway might be a branch line of the ancient stress response regulatory network, and this branch line became specialized for gland structure in Malvaceae. The genome of *G. australe* and its valuable genes and the related regulatory network involved in gossypol/gland formation and disease resistance will be further explored and employed in cotton breeding and sustainable agriculture.

## Discussion

Our study was greatly facilitated by the development of SMRT long‐read sequencing technology. Specifically, this technology can dramatically increase the N50 contig lengths of genome assemblies; we used both BioNanooptical and chromatin interaction mapping approaches in combination with paired‐end sequencing, and this combination worked very well in combination with our long‐read assemblies. After our four‐step assembly process, our final *G. australe* reference genome included 1.75 Gb of sequence, with a scaffold N50 of 143.60 Mb, a contig N50 of 1.83 Mb. Notably, our entire assembly comprised only 650 scaffolds covering the 13 haploid *G. australe* chromosomes. Our high‐quality assembly enabled comparison with the recently published cotton genomes that have been assembled using PacBio data in combination with multiple scaffolding methods (Du *et al*., 2018; Wang *et al*., 2019a).

Based on our new assembly sequencing of *G. australe* genome, we further explored new genes involved in disease resistance and gland formation. Wilt disease caused by *V. dahlia* is the most devastating disease of cotton crops in several parts of the world, including China (Zhang *et al*., 2019). Because the main cultivated upland cotton species (*G. hirsutum*) lacks genetic resources conferring resistance to Verticillium wilt, researchers have surveyed for such resistance genes from relatives such as, *G. barbadense* (Gao *et al*., 2016; Miao *et al*., 2019; Sun *et al*., 2013, 2017; Wang *et al*., 2018a; Xiang *et al*., 2017; Zhang *et al*., 2018; Zhou *et al*., 2018) and other wild species, including *G. australe* (Benbouza *et al*., 2009; Tang *et al*., 2018; Wang *et al*., 2018a,2018c).

However, the molecular mechanism for cotton resistance to Verticillium wilt remains unclear, which has limited progress in developing cotton varieties with resistance to Verticillium (Han *et al*., 2019). Exploring the disease resistance of *G. australe* may facilitate and the genetic improvement of cotton resistance against Verticillium wilt. Our study offers new insights about such molecular mechanisms, in that we empirically demonstrate that expression of the *GauCBP1* and *GauCCD7* gene is induced by *V. dahliae* and by treatment with the plant hormones GR24, SA and MeJA (but not by Eth or ABA). *GauCBP1* knockdown via VIGS markedly reduced cotton resistance to *V. dahliae*, implying that *GauCBP1* functions in the response processes through which *G. australe* resists *V. dahlia* (Figure S16). Calmodulin‐binding proteins (CBPs) are known to transduce calcium signals in response to fungal diseases. The plant‐specific CALMODULIN BINDING PROTEIN 60 (CBP60) protein family includes CBP60a‐g and SYSTEMIC ACQUIRED RESISTANCE DEFICIENT 1 (SARD1) (Bouché *et al*., 2005; Lu *et al*., 2018), virus‐induced silencing of *GhCBP60b* compromised cotton resistance to *V. dahliae*, revealing that CBP60g, SARD1 and GhCBP60b function in *V. dahlia* resistance (Qin *et al*., 2018).

We also found that the carotenoid biosynthesis enzyme CCD7 and the related strigolactone biosynthesis pathway may contribute to Verticillium wilt resistance (Figure S6). Indeed, our VIGS results show that silencing of *GauCCD7* compromised resistance to *V. dahliae* (Figure 4). This is the first report to identify a role for CCD7 against a fungal disease in angiosperms.

Recent studies have reported that genes associated with the strigolactone pathway and with plant architecture function in plant disease resistance (Sun *et al*., 2019; Wang *et al*., 2018b). The tomato mutant *Slccd8* showed increased susceptibility to both pathogens, indicating a new role for strigolactones in plant defence (Torres‐Vera *et al*., 2014). The CCD7 and CCD8 enzymes of the strigolactone pathways were also reported to contribute to resistance against the phytopathogenic fungi in the spreading moss *Physcomitrella patens* (Decker *et al*., 2017). The F‐box protein MAX2 was confirmed to contribute to strigalactone‐associated resistance to bacterial phytopathogens in *Arabidopsis thaliana* (Piisilä *et al*., 2015). Another study reported that a single transcription factor, IPA1 (Ideal Plant Architecture 1), promotes both yield and disease resistance by sustaining a balance between growth and immunity in rice (Wang *et al*., 2018b). Other work has shown that *DWARF14* acts as a receptor for strigolactones in the SL signalling pathway both in rice and cotton (Sun *et al*., 2016; Wang *et al*., 2019b). Overexpression of *Loose Plant Architecture 1* enabled increased planting densities and resistance to sheath blight disease via activation of *PIN‐FORMED 1a* in rice (Sun *et al*., 2019).

In addition, our study identified and cloned a previously unknown gland formation gene: *GauGRAS1*. VIGS silencing of *GauGRAS1* in *G. australe* resulted in a glandless stem and petiole but a glanded leaf, and significantly reduced the gossypol content in the stem and petiole (Figure 4). The expression pattern of *GauGRAS1* was different from that of another known gland development related to gene *GauPGF* (Figure S21) (Figure 4). These findings indicated that *GauGRAS1* may play an important role in delayed morphogenesis of gland morphogenesis in *G. australe*, a distinct role compared to reported functions from other cotton species (Cheng *et al*., 2016; Janga *et al*., 2018; Ma *et al*., 2016).

In conclusion, our work has generated a high‐quality reference genome assembly for a phenotypically distinct diploid wild relative of tetraploid domesticated cotton. Beyond providing a new genomics‐era tool to help cotton improvement programmes increase disease resistance and potentially develop varieties with new combinations of glandless‐seed and glanded‐plant traits, our study also identified multiple genes which we empirically confirmed to function in increasing *G. australe* resistance to fungal infection, findings which should help promote the general use of cotton and the efficiency of cotton production as both a fibre and oilseed crop.

## Methods

### Plant materials and strain selection

DNA samples of *G. australe* were obtained from the Institute of Cotton Research of the Chinese Academy of Agricultural Sciences (accession G2‐lz); the plants showed genetic homozygosity after 15 successive generations of self‐fertilization and were planted in the nursery of the China National Wild Cotton Plantation in Sanya.

Other Australian wild diploid *Gossypium* species with glandless‐seed and glanded‐plant traits were obtained from the Institute of Cotton Research of the Chinese Academy of Agricultural Sciences: these included glanded *G. australe*,* G. nelsonii*,* G. bickii* and Zhongya 1 (*G. arboreum*); few glands in the seeds and glanded plants, such as Xiangmian 18 (*G. hirsutum*); and with glandless seeds and plants, including dominant and recessive glandless Zhongmiansuo 12, as well as *G. raimondii* and Xinhai 15 (*G. barbadense*), C5 (Jinxianduanguozhi, glanded *G. hirsutum*), were used in this research.

### DNA extraction and whole‐genome sequencing

Fresh young leaves of *G. australe* were collected, immediately frozen in liquid nitrogen and stored at −80 °C until DNA extraction. A standard phenol–chloroform method was used for DNA extraction with RNase A and proteinase K treatment to prevent RNA and protein contamination. Genomic DNA was sheared to a size range of 15–40 kb, enzymatically repaired and converted into SMRTbell template libraries as recommended by Pacific Biosciences. The resulting SMRTbell templates were sequenced on a PacBio Sequel instrument. A total of 18 SMRT cells were sequenced producing 82 Gb SMRT raw data. Genomic DNA was used to construct five paired‐end libraries with insert sizes (in KB) of 0.5, 0.8, 2k, 5k and 10k, using a Paired‐End DNA Sample Prep kit (Illumina). These libraries were sequenced using the Illumina HiSeq Xten platform, producing 151G, 138G, 78G, 75G and 77G raw data, respectively.

### *De novo* assembly of the genome using PacBio and Illumina data

Primary contigs were assembled from PacBio long reads by MECAT (version 1.0) (Xiao *et al*., 2017). Overlaps of long reads were found using the command mecat2pw (parameters: ‐k 4 –a 2000) and were corrected using the command mecat2cns (parameters: ‐r 0.9 –c 6 –l 5000). The 25× coverage of the longest corrected reads was extracted and assembled using the command mecat2canu (min Overlap Length = 500, min Read Length = 1000). The resulting contigs were polished using more than 100× coverage of Illumina short reads by Pilon (version 1.22) with default parameters (Walker *et al*., 2014). A total of 32 651 SNPs and 1 029 916 InDels were detected and corrected. SSPACE (version 3.0) was used (with default settings) to join contigs to scaffolds as follows: The large‐insert read pairs are mapped against the pre‐assembled PacBio contigs using Bowtie (version 1.1.1). The position and orientation of each pair that could be mapped is stored in a hash. After removing duplicate read pairs‐pairs, scaffolds are formed by iteratively combining contigs if a minimum number of read pairs (*k* = 5) support the connection, starting with the largest contig. Scaffolds are extending in the same way direction until either a contig has no links with other contigs.

### BioNano Genomics DLS optical maps to improve genome assemblies

Optical maps were *de novo* assembled into genome maps using BioNano assembler software (Solve System, BioNano Genomics). Single molecules longer than 150 kb with at least 8 fluorescent labels were used to find possible overlaps (*P *< 1 × 10^−10^). The BioNano Solve software imports the assembly and identifies putative nick sites in the sequence based on the nicking endonuclease‐specific recognition site. These *in silico* maps for the sequence contigs were then aligned to the *de novo* BioNano genome maps. Genome maps orient contigs and size gaps by bridging across repeats and other complex elements that break the NGS/TGS assemblies. A total of 884 conflicts between the two are identified and resolved, and hybrid scaffolds are generated in which sequence maps are used to bridge BioNano maps and vice versa. Finally, the sequence assembly corresponding to this hybrid scaffold was generated.

### Hi‐C assembly

We constructed Hi‐C fragment libraries with 300–700 bp insert sizes as described in Rao *et al*. (2014) and sequenced them with an Illumina platform. The clean Hi‐C reads were first truncated at the putative Hi‐C junctions, and then, the resulting trimmed reads were realigned to the assembly results with a BWA aligner (Li and Durbin, 2009). Only uniquely aligned pair reads whose mapping quality was more than 20 were used for further analysis. Invalid read pairs, including dangling‐end, self‐cycle, relegation and dumped products, were filtered by HiC‐Prov2.8.1 (Servant *et al*., 2015). The unique mapped read pairs were valid interaction pairs and were used for scaffolds clustered, ordered and orientated onto chromosomes by LACHESIS (Burton *et al*., 2013). The final pseudo‐chromosomes were constructed after manual adjustment.

### Annotation of TEs

Tandem Repeats Finder (Benson, 1999) was used to search the genome for tandem repeats. Both *de novo* and homology‐based approaches were used to find TEs. Programmes including RepeatProteinMask and RepeatMasker (Tarailo‐Graovac and Chen, 2009) were applied to identify TEs through commonly used databases of known repetitive sequences, and Repbase was used along with a database of plant repeating sequences and our *de novo* TE library to find repeats with RepeatMasker (Jurka *et al*., 2005). Intact LTRs were predicted using LTR_STRUC (McCarthy and McDonald, 2003). The insert time of all intact LTRs was calculated with the formula: time = Ks/2r (Ks is synonymous substitutions per synonymous site, and r is the rate of nucleotide substitution, which was set to 7 × 10^−9^).

### Gene prediction

The MAKER pipeline (Campbell *et al*., 2014) was used to annotate protein‐coding genes, integrating *ab initio*‐predicted genes including analysis of AUGUSTUS (Stanke *et al*., 2006), SNAP (Korf, 2004) and GeneMark (Borodovsky and Lomsadze, 2011); 149 916 *Gossypium* unigenes downloaded from the cottongen website ( https://www.cottongen.org/), *de novo* assembled transcripts from short‐read mRNA sequencing (mRNA‐seq) in this research, and proteins from *A. thaliana*,* Theobroma cacao*, and *Durio zibethinus*. Transposons and low‐confidence predictions were removed.

### Gene family and phylogenetic analysis

All‐versus‐all BLASTP (E value < 1 × 10^−7^) comparison of all protein sequences for eight species (*G. arboreum*,* G. raimondii*,* G. australe*,* Glycine max*,* Dimocarpus longan*,* T. cacao*,* Cucurbita maxima*,* D. zibethinus*) was performed, and orthologous genes were clustered by OrthoMCL (Li *et al*., 2003). CAFE was applied to identify gene families that had undergone expansion and/or contraction (De Bie *et al*., 2006).

Single‐copy gene families were used to construct a phylogenetic tree. MUSCLE (Edgar, 2004) was used to generate a multiple sequence alignment of protein sequences for each single‐copy family with default parameters. The alignments of each family were concatenated to a super alignment matrix that was used for phylogenetic tree reconstruction through maximum likelihood (ML) methods. The divergence time between species was estimated using MCMC tree in PAML (Yang, 1997) with the options ‘correlated molecular clock’ and ‘HKY85’ model. A Markov Chain Monte Carlo analysis was run for 1 000 000 generations using a burn‐in of 100 000 iterations. Divergence time for the root node of Malvaceae obtained from the fossil estimate (Carvalho *et al*., 2011; Grover *et al*., 2017) and TimeTree database ( http://www.timetree.org/) was used as the calibration point.

### Whole‐genome duplication analysis and whole‐genome alignment

We used MCScan (Tang *et al*., 2008) to identify syntenic blocks and calculate Ks rates for syntenic genes. For analysis of the WGD of *G. australe*,* G. arboreum* and *G. raimondii*, paralogous gene pairs originating from their respective WGDs were identified, and the Ks value of each gene pair was calculated. After the repeat regions were masked, whole‐genome alignment was carried out by LASTZ between *G. australe* and *G. raimondii* and between *G. australe* and *G. arboreum*.

### PSG analysis

Based on the aforementioned phylogenetic tree, the branch‐site model incorporated in the PAML package was used to detect PSGs (Zhang *et al*., 2005). For the detection of PSGs in *G. australe*, the branch of *G. australe* was used as the foreground branch, and all other branches in the phylogenetic tree were used as background branches. Similar approaches were used to detect PSGs in *G. arboreum* and *G. raimondii*.

### Transcriptome analysis

RNA‐seq reads were mapped to the reference genome using TopHat (Trapnell *et al*., 2009). To measure the gene expression level in tissues, we calculated the expression of genes using FPKM (fragments per kilobase of exon model per million mapped reads) with Cufflinks (Trapnell *et al*., 2010). To identify differentially expressed genes across samples or groups, we used the edgeR package ( http://www.rproject.org/). We defined genes with a fold change ≥ 2 and a false discovery rate (FDR) < 0.05 in comparison as significant DEGs. The DEGs were then subjected to enrichment analysis of GO functions and KEGG pathways.

### Inoculation of *Verticillium dahliae* V991

For treatment with *V. dahliae*, a highly aggressive defoliating fungus, *V. dahliae* V991, was incubated on a potato‐dextrose agar plate for 1 week and then inoculated into Czapek broth on a shaker at 120 rpm at 25°C for 3–4 days until the concentration of spores reached approximately 10^8^–10^9^ spores (mL^−1^). The suspension liquid was adjusted to 10^7^ spores (mL^−1^) with sterile distilled water for inoculation (Xu *et al*., 2011). The seeds of *G. australe*,* G. arboreum* and *G. raimondii* were grown in commercial sterilized soil at 24°C/20°C day/night temperatures with a photoperiod of 14‐h light and 10‐h dark for 2–3 weeks and a relative humidity of 60%. The cotton seedlings of 2 true leaves were infected with *V. dahliae* by root dip inoculation into a suspension of fungal spores for 1 min and then returned to their original pots. Control plants were not inoculated but were otherwise treated and were mock inoculated using distilled water in the same way. Whole cotton plants were harvested for sample preparation at 24‐h, 48‐h and 72‐h postinfection time points.

### Seed germination experiment

Thirty seeds of *G. australe*,* G. arboreum* and *G. hirsutum* were delinted. The seeds were soaked in distilled water for 5 h, and the outer seed coat was peeled off and then soaked in distilled water for 1 h to remove the inner seed coat. Next, the seeds were covered with moist cotton wool and germinated under dark conditions (28°C). The germinating seeds were collected at 8 h (before gland formation) and 22–46 h for different species (at the beginning stage of gland formation) for transcriptome analysis (Figure 1). Finally, the germinated seeds were taken, photographed under a stereomicroscope, or rapidly frozen with liquid nitrogen prior to extraction of total RNA.

### Virus‐induced gene silencing assay

For knockdown of *GauPGF*,* GauGRAS1*,* GauCCD7* and *GauCBP1*, approximately 300‐bp fragments of the target genes were PCR‐amplified from *G. australe* cDNA. The primers used were V‐*GauPGF*‐F and V‐*GauPGF*‐R, V‐*GauGRAS1*‐F and V‐*GauGRAS1*‐R, V‐*GauCCD7*‐F and V‐*GauCCD7*‐R, and V‐*GauCBP1*‐F and V‐*GauCBP1*‐R (Table S12). The PCR products were cloned into pTRV2 to produce the VIGS vectors TRV:GauPGF, TRV:GauGRAS1, TRV:GauCCD7 and TRV:GauCBP1. The pTRV1 and recombinant pTRV2 vectors were introduced into the *Agrobacterium* strain GV3101. *Agrobacterium* strains harbouring the TRV:GauPGF, TRV:GauGRAS1, TRV:GauCCD7 and TRV:GauCBP1 plasmids combined with strains harbouring the pTRV1 vector were mixed in a 1:1 ratio. Seedlings with mature cotyledons but without a visible rosette leaf (14 days after germination) were infiltrated by inserting the *Agrobacterium* suspension into the cotyledons via a syringe (the *GauCCD7* and *GauCBP1* gene silencing using VIGS was done in Xinhai‐15 (*G. barbadense*), and *GauPGF* and *GauGRAS1* gene silencing was done in *G. australe*). The plants were grown at 23°C with a 16‐h light and 8‐h dark cycle and 80% humidity. The effectiveness of the VIGS assay was detected by generating the TRV:GbCLA construct using the *G. barbadense* CLA1 gene as previously described. TRV:00 was used as a control vector.

### Identification of Verticillium wilt resistance of VIGS cotton

The VIGS (TRV:GauCCD7, TRV:GauCBP1) plants and the control (TRV:00) plants were inoculated with a *V. dahliae* conidia suspension by injuring the roots, and the Verticillium wilt symptoms were investigated and compared at 17 days postinfection. The rate of diseased plants was determined from approximately 30 seedlings per treatment, and the assessment was repeated at least three times. The DI was calculated according to the following formula: DI = [(∑disease grades × number of infected plants)/(total examined plants × 4)] × 100%] (Hu *et al*., 2018). The DI was scored using at least 25 plants per treatment and repeated at least three times.

### Quantitative RT‐PCR analysis

Different tissues of the cotton plants, including ovules, roots, stems and leaves, were collected. Total RNA was extracted and then reverse transcribed into cDNA. The *GbUBQ7* gene was selected as an internal reference gene, and Primer Premier 6.0 software was used to design specific quantitative primers (q‐*UBQ7*‐F, q‐*UBQ7*‐R; q‐*GauPGF*‐F, q‐*GauPGF*‐R; q‐*GauGRAS1*‐F, q‐*GauGRAS1*‐R; q‐*GauCCD7*‐F, q‐*GauCCD7*‐R; q‐*GauCBP1*‐F, q‐*GauCBP1*‐R) (Table S12). The experiment was performed using a Roche LightCycler 480 Real‐Time PCR System with Q711‐ChamQ Universal SYBR qPCR Master Mix (Vazyme, Nanjing, China); the reaction procedure was 40 cycles of 95°C for 30 s, 95°C for 10 s and then 60°C for 30 s.

### Gossypol content analysis

Leaves and stems were taken separately from the plants to measure the free gossypol content, which was measured by spectrophotometric methods with phloroglucinol, as described previously (Gao *et al*., 2013). Briefly, each 100 mg plant sample was ground into powder with liquid nitrogen. Then, 0.5 mL extract (acetonitrile/water = 80:20) was added, and the samples were oscillated at 4°C for 45 min. The extract was centrifuged at 12 000 *g* for 15 min. The supernatant was carefully transferred into a new EP tube. Finally, a double volume of phloroglucinol (Solarbio) chromogenic solution was added, and the samples were incubated in a 55°C water bath for 5 min. The sample was analysed at a wavelength of 550 nm. A gossypol reference standard as purchased from the website www.biaowu.com.

### Histochemistry and microscopy

A fixative (1 mL 5% glutaraldehyde+4% paraformaldehyde) was added to a 2.0‐mL centrifuge tube. The materials (leaf, stem, petiole) were rapidly cut into 1‐2 mm with a sharp blade, immediately placed into the fixative and incubated overnight at room temperature. Then, the fixative was aspirated, 1 mL 0.1 m phosphate buffer was added, and the sample was rinsed twice (15 min each time). Next, the rinse solution was aspirated, and the samples were dehydrated with 30%, 50%, 70%, 90% and 100% ethanol for 30 min each time. The reagent (100% ethanol: LR White embedding agent = 1:1) was added for 1 h, and the pure embedding agent was added for 5 h. The sample was then soaked in a pure embedding agent overnight. Finally, the sample was embedded in a capsule (polymerization in a 60°C incubator for 12 h). The embedding blocks were formed. Semithin sections (1–2 μm) were cut with a Leica‐UC7 ultrathin microtome and stained with 0.05% crystal violet (a drop of 0.05% crystal violet stain was added to the section, the dye solution was immediately drained, and the excess dye solution was rinsed away with distilled water). The samples were observed and photographed under a microscope.

## Author contributions

F.L., Y.F.C., X.Z. and K.B.W. designed and conceived the research programme. F.L., X.Y.C., K.B.W., Z.L.Z., Q.L.W., B.L.Z. and R.H.P. prepared samples of DNA sequencing and RNA‐seq. P.W., C.X.W., S.P.G., B.L., Q.D., Y.Q., J.R.C., Y.Y.Z., Q.S., Y.H.X., Y.C. and X.Y.S. performed gene function analyses. L.Y.M. performed histochemistry and microscopy analyses. Y.C. performed TE insertion time analysis, phylogenetic tree analysis and positively selected genes analysis. S.M.H performed the GO term enrichment and gene family expansion analysis, whole‐genome duplication analysis and collinearity analysis. M.S.S. was involved in the transcriptome analyses. C.W.C., Z.L., F.F.Y., Y.Z. and Y.C.X. involved in bioinformatics analyses. Y.Z. and P.A. directed the genome sequencing and assembly parts of the project. F.F.H. performed genome and transposable elements (TEs) annotation. F.L., Y.F.C., G.Y.A., K.B.W. and X.Z. discussed results. Y.F.C. and F.L. contributed to the writing the main text of the manuscript. F.L., Y.F.C., X.Z., G.Y.A. and K.B.W. reviewed the final version.

## Competing Interests

The authors declare there are no competing interests.

## Supporting information


**Figure S1** Illustration of misassemblies in the genome of *G. australe* examined using BioNano optical maps.
**Figure S2** Interaction frequency distribution of Hi‐C links among chromosomes.
**Figure S3** K‐mer analysis for estimating the genome size of *G. australe*.
**Figure S4** Venn diagram analyses of unique and conserved genes or gene families.
**Figure S5** Syntenic blocks between *G. australe* and *G. arboreum* genome (Left), *G. australe* and *G. raimondii* genome (Right).
**Figure S6** Venn graph of genes subjected to positive selection in *G. australe*,* G. arboreum* and *G. raimondii*.
**Figure S7** Enriched pathway of the PSGs in *G. australe* and *G. arboreum*.
**Figure S8** Expression levels of *CCD7* in different tissues of three diploid cotton species.
**Figure S9** Expression levels of *GauCCD7* in different tissues of *G. australe* plant treated with Verticillium *dahliae*.
**Figure S10** Expression levels of *GauCCD7* in CSSL‐1 seedlings treated with different hormones.
**Figure S11** Trend analysis of differently expressed genes response to Verticillium wilt in *G. australe*.
**Figure S12** Venn diagram analyses of up‐regulated profile genes in *G. australe* and down‐regulated profile genes in *G. arboreum*.
**Figure S13** Expression levels of *CBP1* in different tissues of three diploid cotton species.
**Figure S14** Expression levels of *GauCBP1* in different tissues of *G. australe* plant treated with Verticillium *dahliae*.
**Figure S15** Expression levels of *GauCBP1* in CSSL‐1 seedlings treated with different hormones.
**Figure S16** The silencing of *GauCBP1* from *G. australe* compromised cotton resistance to *V. dahliae* in Xinhai 15.
**Figure S17** Expression of *GauGRAS1 and GauPGF* in ovules of different gland materials.
**Figure S18** Relative expression level of *GauPGF* and *GauGRAS1* gene in leaves (adult stage) and ovules (10 dpa) in two diploid G subgroup wild cotton species by qRT‐PCR.
**Figure S19** Relative expression level of *GoPGF* and *GRAS1* gene in leaves and ovules in three diploid G subgroup wild cotton species, 16 dpa.
**Figure S20** Relative expression level of *PGF* and *GRAS1* gene before and after GF (gland formation) during seed germination of three cotton species.
**Figure S21** Functional characterization of *GauPGF* by VIGS.
**Figure S22** Module‐trait relations. Each row corresponds to a module eigengene, column to a transcriptome.
**Figure S23** Local collinearity based on forty genes adjacent to *GoPGF* of two example species.
**Figure S24** Local collinearity of *GoPGF* between *G. hirsutum* and other genomes of angiosperm selected (blue) from the Angiosperm Phylogeny Group (APG) IV system.
**Figure S25** Local collinearity of the *GRAS1* gene between *G. hirsutum* and other genomes of angiosperm selected (blue) from the Angiosperm Phylogeny Group (APG) IV system.

**Table S1** Genome assembly statistics for *G. australe*.
**Table S2** Assessment of sequence coverage of the *G. australe* genome assembly by homologous search using full‐length transcripts.
**Table S3** Number of genes with homology or functional classifications by different methods.
**Table S4** Analysis of non‐coding RNA genes in the *G. australe* genome.
**Table S5** Evaluation the quality of the annotation using the BUSCO method.
**Table S6** Summary and content analysis of different types of TEs in the *G. australe* genome.
**Table S7** Analysis of the content of major TE subfamilies in three *Gossypium* genomes.
**Table S8** Relative distribution (%) of Gypsy retrotransposon subgroups in the genomes of three *Gossypium* genomes.
**Table S9** Annotation of 31 genes that were up‐regulated profile genes in *G. australe* and down‐regulated profile genes in *G. arboreum*.
**Table S10** Annotation of 10 hub genes with top ten connectivity the magenta4 module of WGCNA analysis.
**Table S11** Annotation of genes adjacent to GoPGF co‐expressed in magenta4 module.
**Table S12** Primers used in VIGS and qRT‐PCR.

**Data S1** PSGs in *G. australe*,* G. arboreum* and *G. raimondii*.

## Data Availability

The raw sequencing data reported in this paper have been deposited at in the NCBI BioProject database under accession number PRJNA513946. This Whole Genome Shotgun project has been deposited at DDBJ/ENA/GenBank under the accession SMMG00000000. The version described in this paper is version SMMG01000000.
