# Supplementary material for: Genome sequencing of the Australian wild diploid species Gossypium australe highlights disease resistance and delayed gland morphogenesis
Source: Plant Biotechnol J. 2019 Sep 13;18(3):814–28. doi: 10.1111/pbi.13249 (PMC7004908; doi:10.1111/pbi.13249)
Supplement: Supplementary file 1 — Figure S1 Illustration of misassemblies in the genome of G. australe examined using BioNano optical maps. Figure S2 Interaction frequency distribution of Hi‐C links among chromosomes. Figure S3 K‐mer analysis for estimating the genome size of G. australe. Figure S4 Venn diagram analyses of unique and conserved genes or gene families. Figure S5 Syntenic blocks between G. australe and G. arboreum genome (Left), G. australe and G. raimondii genome (Right). Figure S6 Venn graph of genes subjected to positive selection in G. australe, G. arboreum and G. raimondii. Figure S7 Enriched pathway of the PSGs in G. australe and G. arboreum. Figure S8 Expression levels of CCD7 in different tissues of three diploid cotton species. Figure S9 Expression levels of GauCCD7 in different tissues of G. australe plant treated with Verticillium dahliae. Figure S10 Expression levels of GauCCD7 in CSSL‐1 seedlings treated with different hormones. Figure S11 Trend analysis of differently expressed genes response to Verticillium wilt in G. australe. Figure S12 Venn diagram analyses of up‐regulated profile genes in G. australe and down‐regulated profile genes in G. arboreum. Figure S13 Expression levels of CBP1 in different tissues of three diploid cotton species. Figure S14 Expression levels of GauCBP1 in different tissues of G. australe plant treated with Verticillium dahliae. Figure S15 Expression levels of GauCBP1 in CSSL‐1 seedlings treated with different hormones. Figure S16 The silencing of GauCBP1 from G. australe compromised cotton resistance to V. dahliae in Xinhai 15. Figure S17 Expression of GauGRAS1 and GauPGF in ovules of different gland materials. Figure S18 Relative expression level of GauPGF and GauGRAS1 gene in leaves (adult stage) and ovules (10 dpa) in two diploid G subgroup wild cotton species by qRT‐PCR. Figure S19 Relative expression level of GoPGF and GRAS1 gene in leaves and ovules in three diploid G subgroup wild cotton species, 16 dpa. Figure S20 Relative expression [file PBI-18-814-s002.docx]

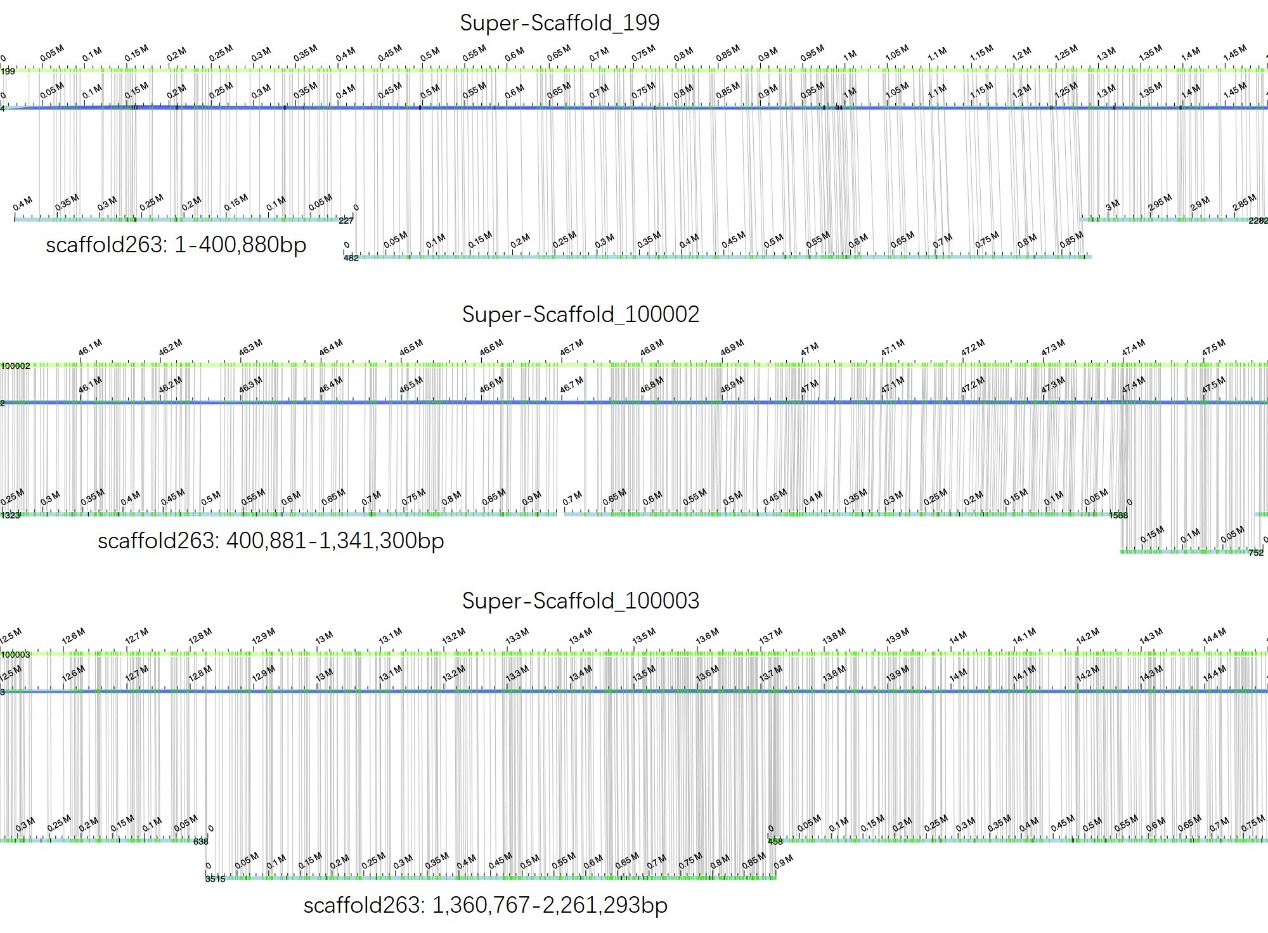


**Supplementary Fig 1. Illustration of misassemblies in the genome of *G.australe* examined using BioNano optical maps**. Scaffold263 assembled by pacbio reads was conflict with BioNano map, which was corrected into four parts, assigned to Super-Scaffold_199, Super-Scaffold_100002 and Super-Scaffold_100003, respectively. Another part was not assigned to any Super-Scaffold.


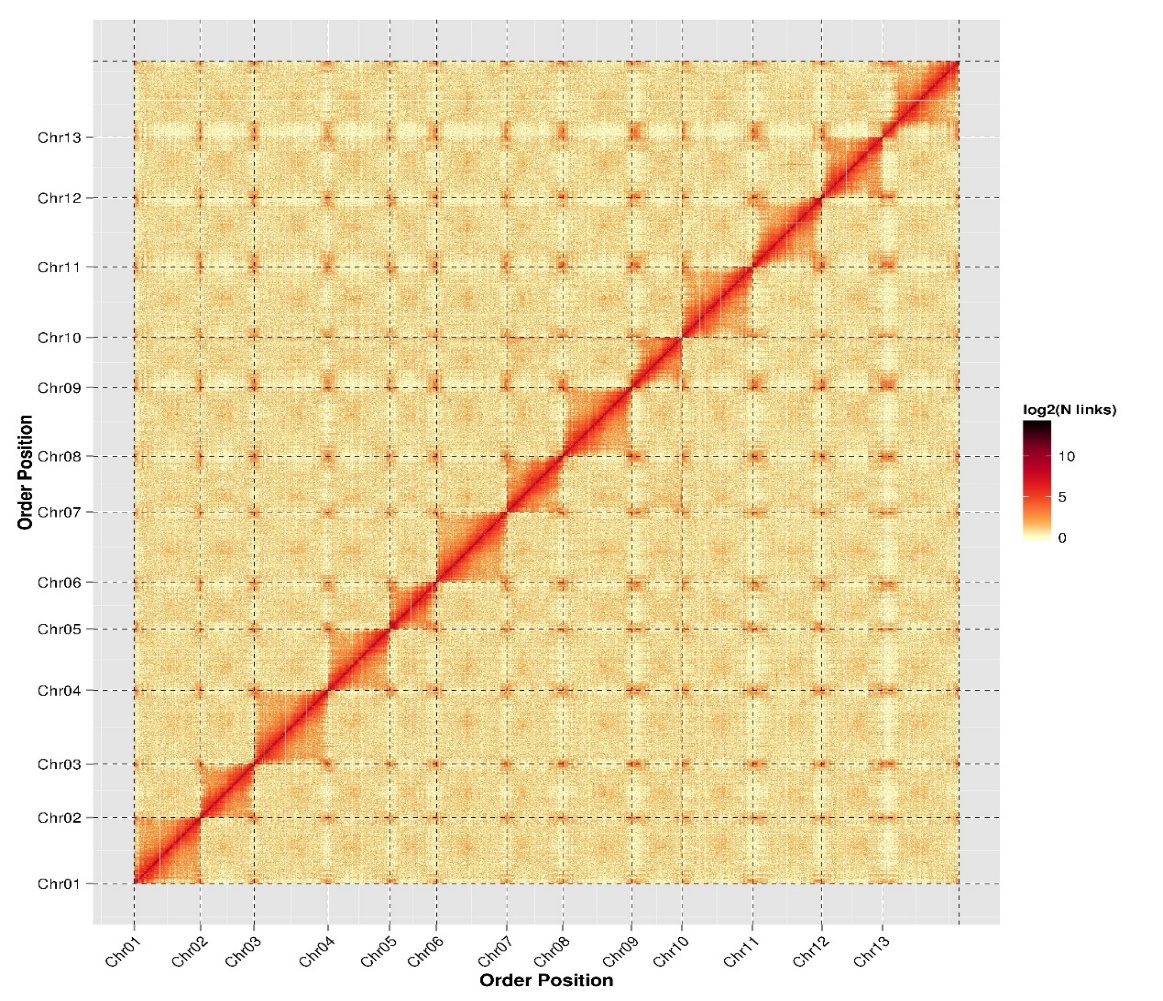


**Supplementary Fig 2. Interaction frequency distribution of Hi-C links among chromosomes.** We scanned the genome by 100-kb nonoverlapping window as a bin and calculated valid interaction links of Hi-C data between any pair of bins. The log2 of link number was calculated. The distribution of links among chromosomes was exhibited by heatmap. The color key of heatmap ranging from light yellow to dark red indicated the frequency of Hi-C interaction links from low to high.

**
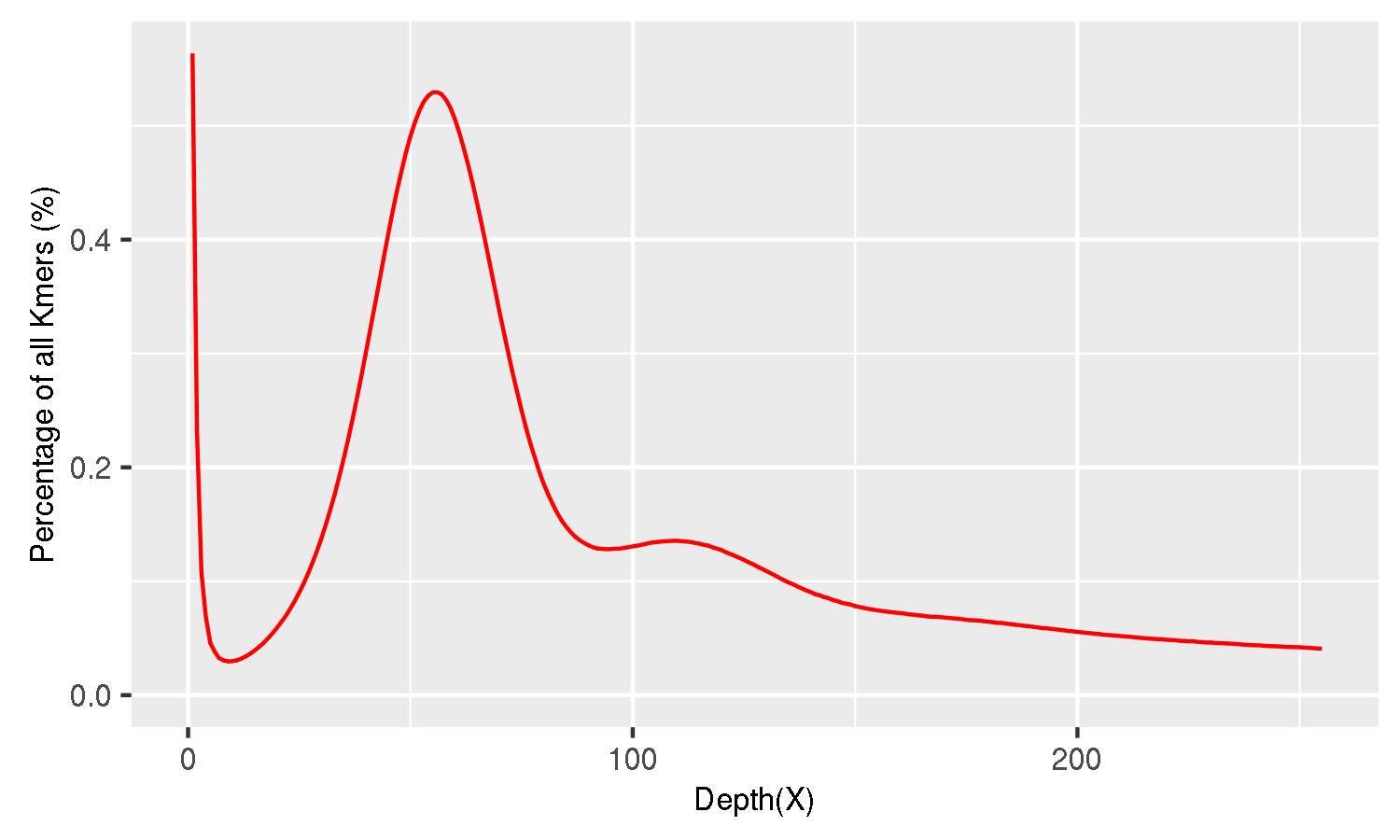
**

**Supplementary Fig. 3. K-mer analysis for estimating the genome size of *G. australe***. The genome size was estimated to be 1,669 Mb.


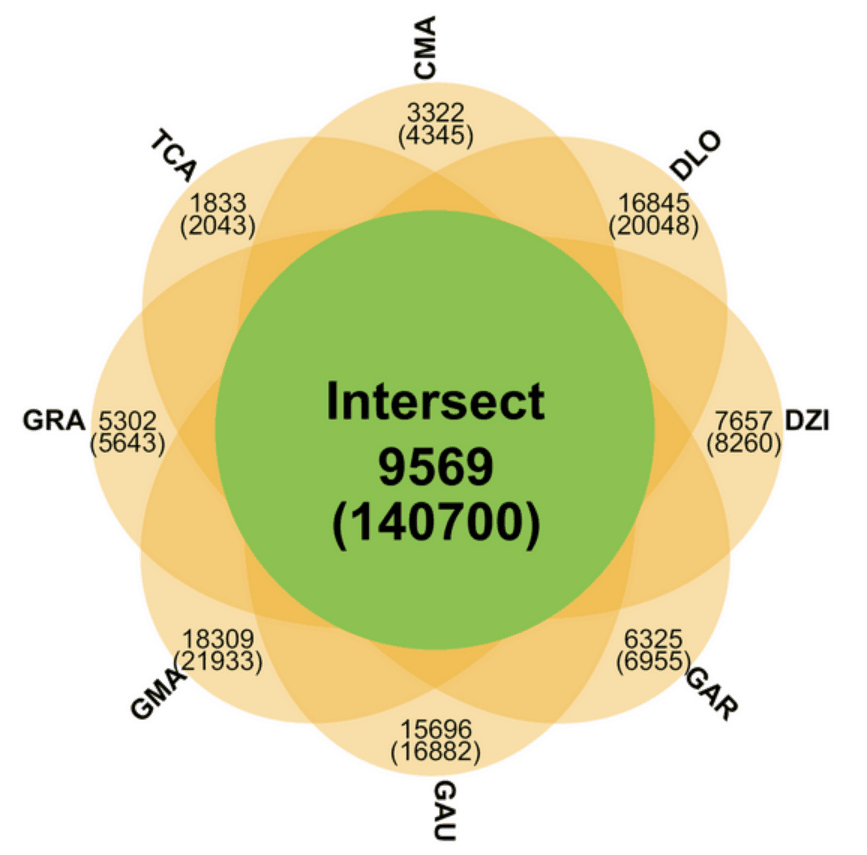


**Supplementary Fig. 4. Venn diagram analyses of unique and conserved genes or gene families.** Among including *Gossypium arboreum* (GAR), *Gossypium raimondii* (GRA), *Gossypium australe* (GAU), *Glycine max* (GMA), *Dimocarpus longan* (DLO), *Theobroma cacao* (TCA), *Cucurbita maxima* (CMA), *Durio zibethinus* (DZI) defined by OrthoMCL.


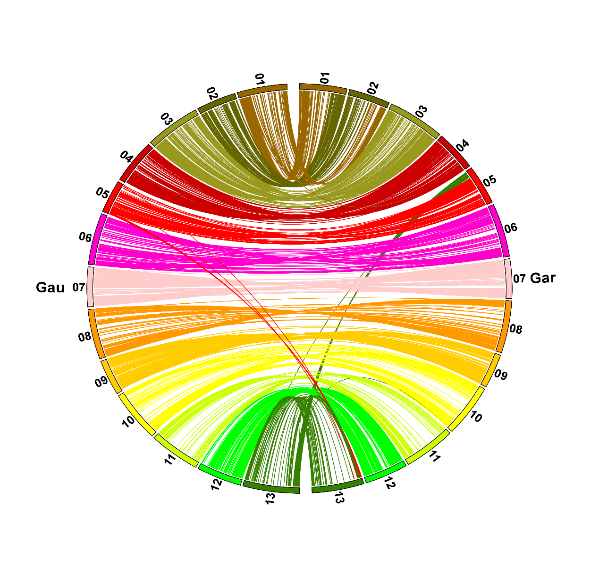

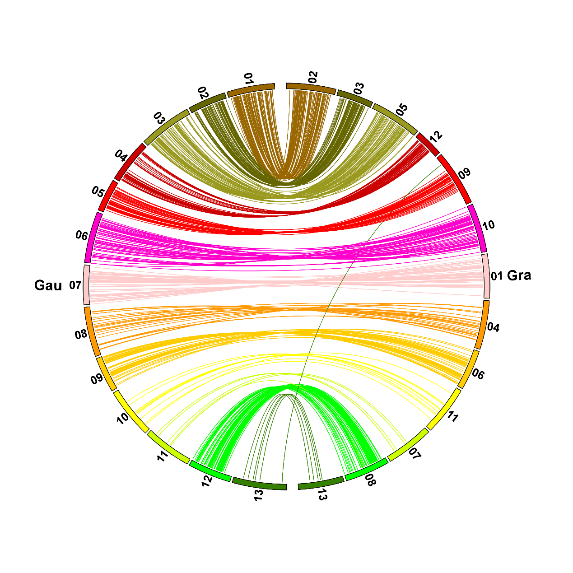


**Supplementary Fig 5. Syntenic blocks between *G. australe* and *G. arboreum* genome (Left), *G. australe* and *G. raimondii* genome (Right)**. Only syntenic blocks of >100 kb in length are shown.


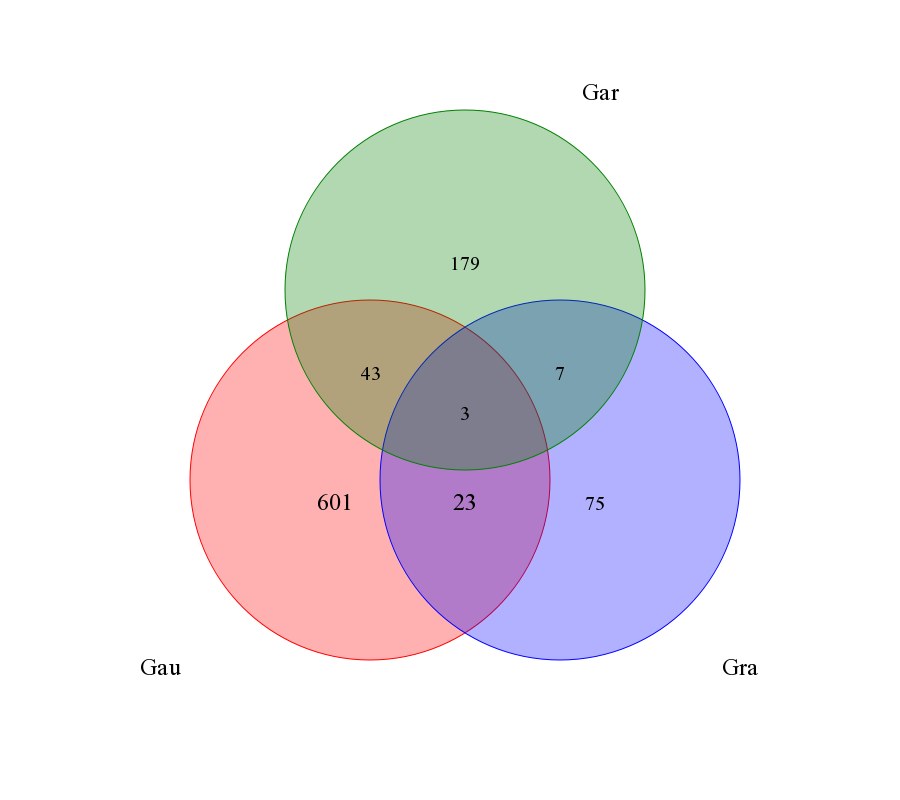


**Supplementary Fig. 6. Venn graph of genes subjected to positive selection in *G. australe*, *G. arboreum* and *G. raimondii*.**


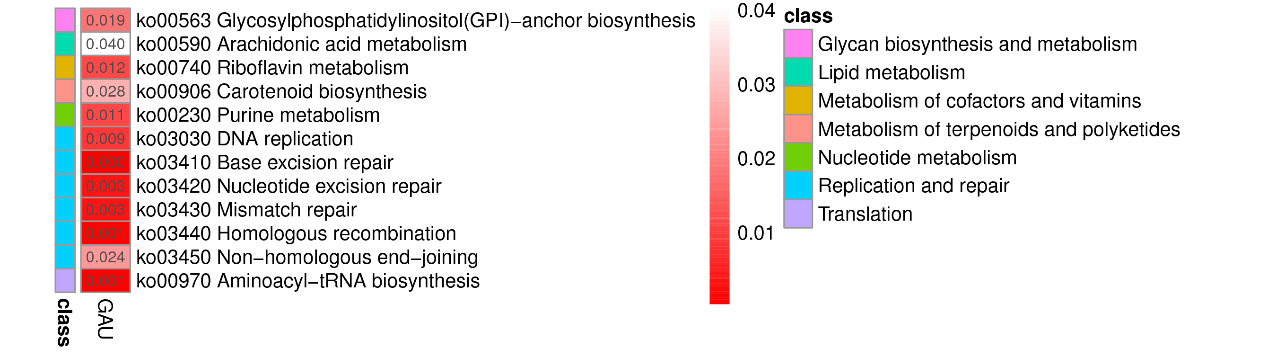


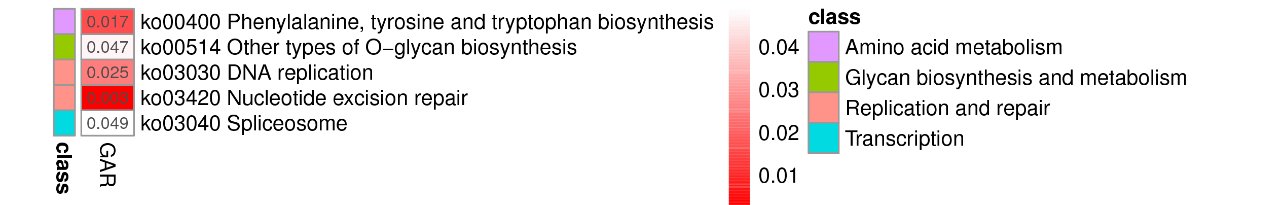


**Supplementary Fig. 7. Enriched pathway of the PSGs in *G. australe* and *G. arboreum*.** Upper: *G. australe*, lower: *G. arboreum*.


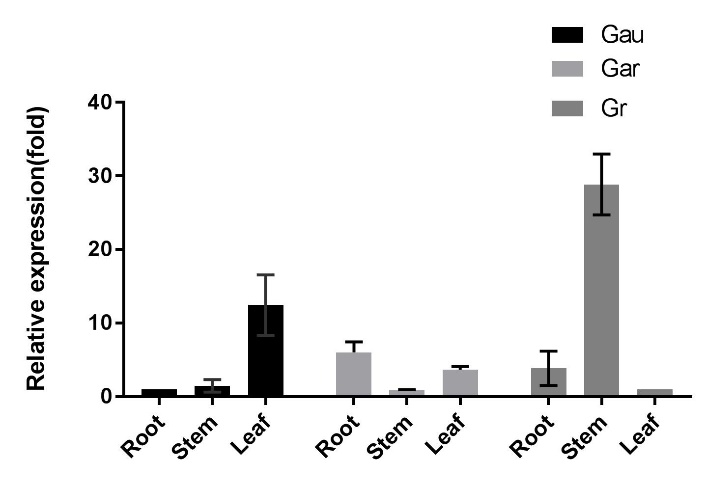


**Supplementary Fig. 8. Expression levels of *CCD7* in different tissues of three diploid cotton species.** Tissues selected were roots, stems, and leaves of *G. australe* (G-genome)*, G. arboretum* (A-genome)*, G. raimondii* (D-genome) seedlings (20-day).


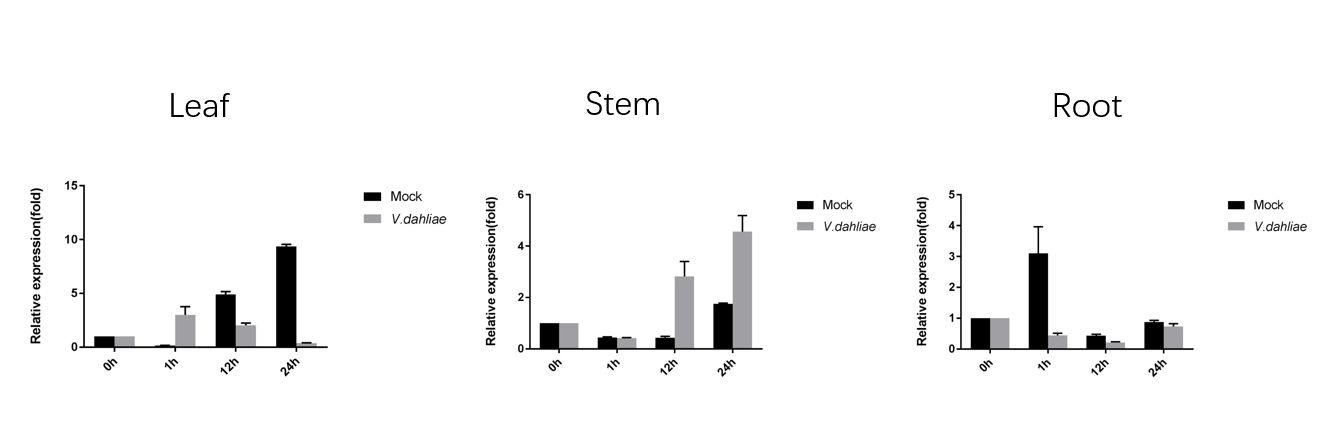


**Supplementary Fig. 9. Expression levels of *GauCCD7* in different tissues of *G. australe*** **plant treated with *Verticillium dahliae*.** Tissues selected were roots, stems, and leaves of *G. australe* seedlings (40-day) after treated with *Verticillium dahliae* 0 h, 1 h, 12 h, 24 h.


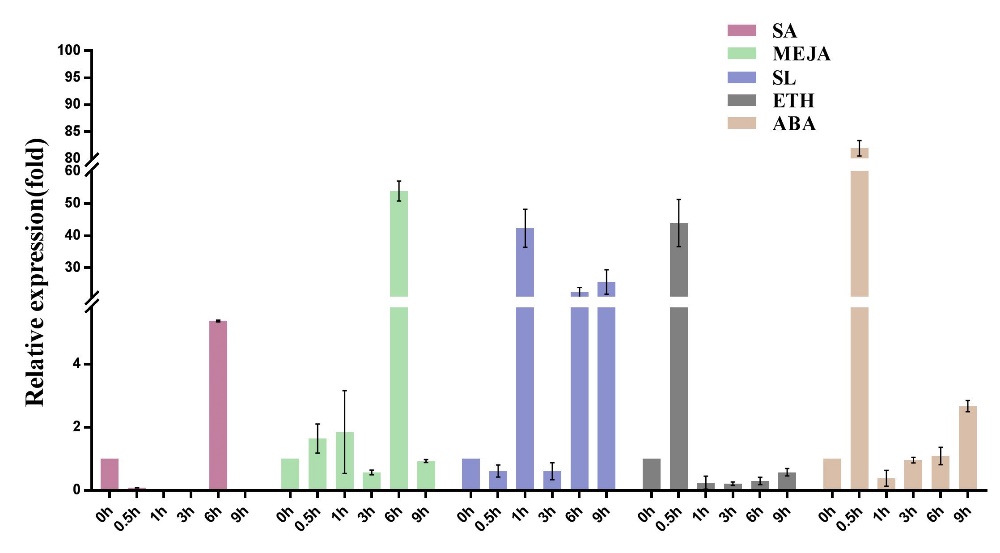


**Supplementary Fig. 10. Expression levels of *GauCCD7* in CSSL-1 seedlings treated with different hormones.** Tissues selected were true leaves of CSSL-1 seedlings (21-day) after sprayed with SA (5 mM), MEJA (100 μM ), SL (5 μM), ETH (100 μM ) and ABA (50 μM ) 0 h, 0.5 h, 1 h, 3 h, 6 h, 9 h.


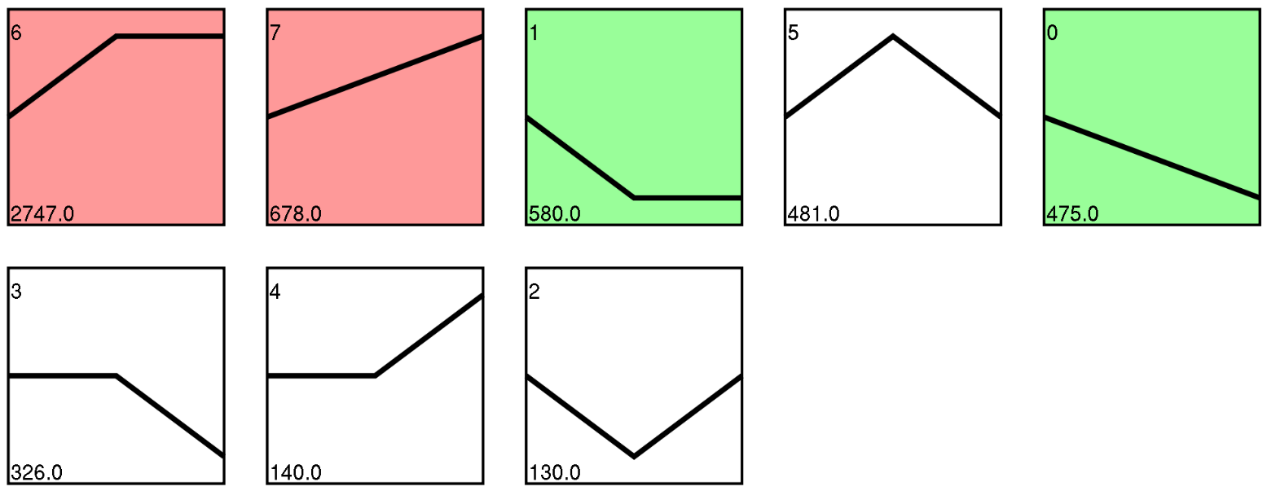


**Supplementary Fig. 11. Trend analysis of differently expressed genes response to Verticillium wilt in *G. australe*.** Profile 6 and profile 7 were the most significant two modules.


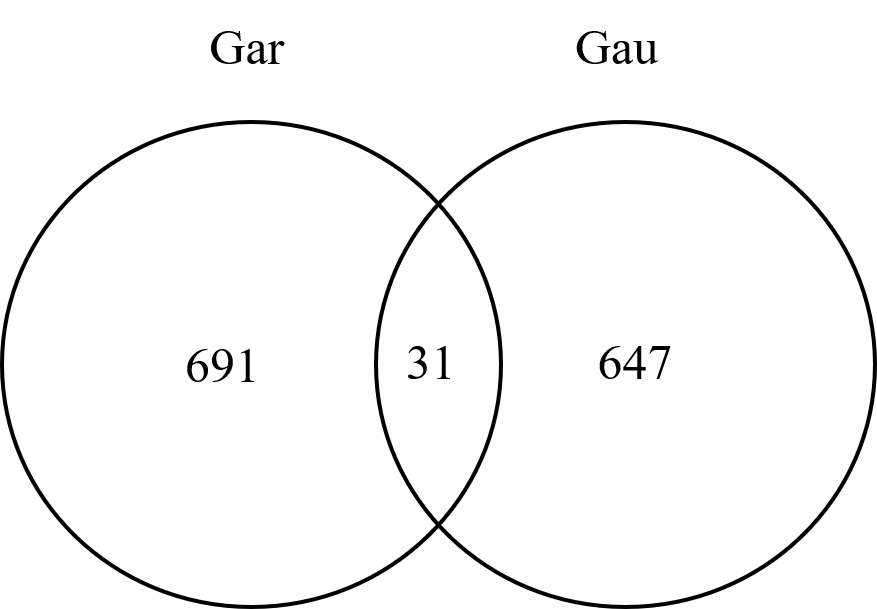


**Supplementary Fig. 12. Venn diagram analyses of up-regulated profile genes in *G. australe* and down-regulated profile genes in *G. arboreum*.**


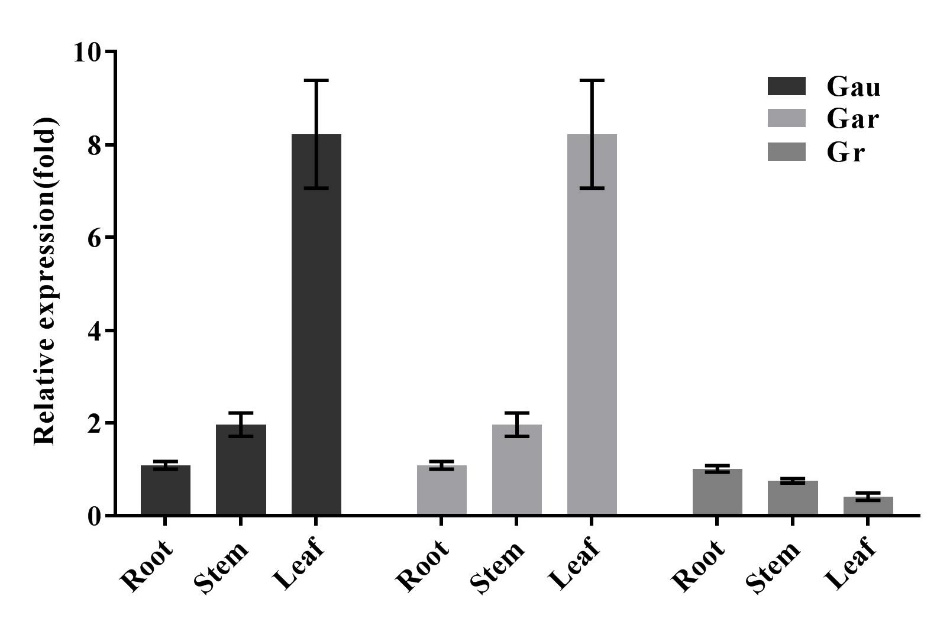


**Supplementary Fig. 13. Expression levels of *CBP1* in different tissues of three diploid cotton species.** Tissues selected were roots, stems, and leaves of *G. australe* (G-genome)*, G. arboretum* (A-genome)*, G. raimondii* (D-genome) seedlings (20-day).


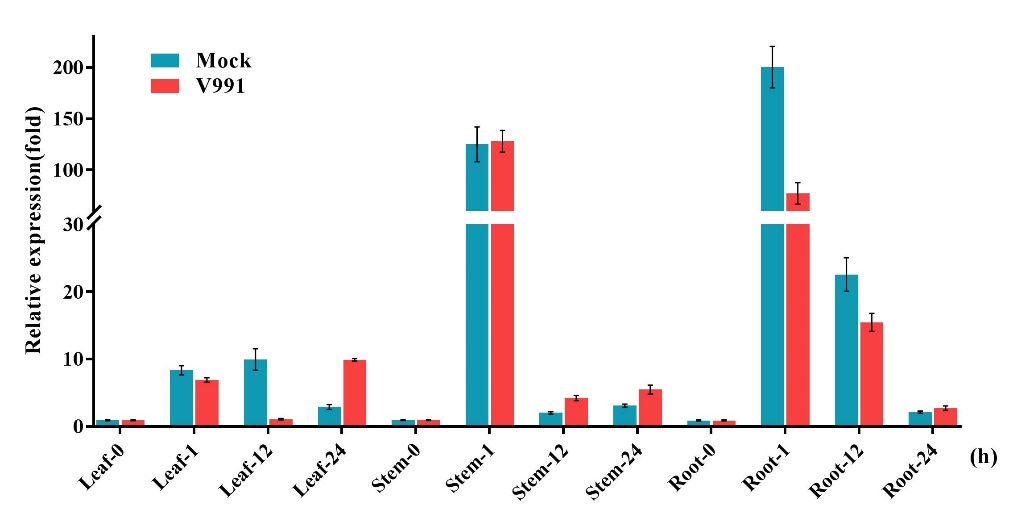


**Supplementary Fig. 14. Expression levels of *GauCBP1* in different tissues of *G. australe*** **plant treated with *Verticillium dahliae*.** Tissues selected were roots, stems, and leaves of *G. australe* seedlings (40-day) after treated with *Verticillium dahliae* 0 h, 1 h, 12 h, 24 h.


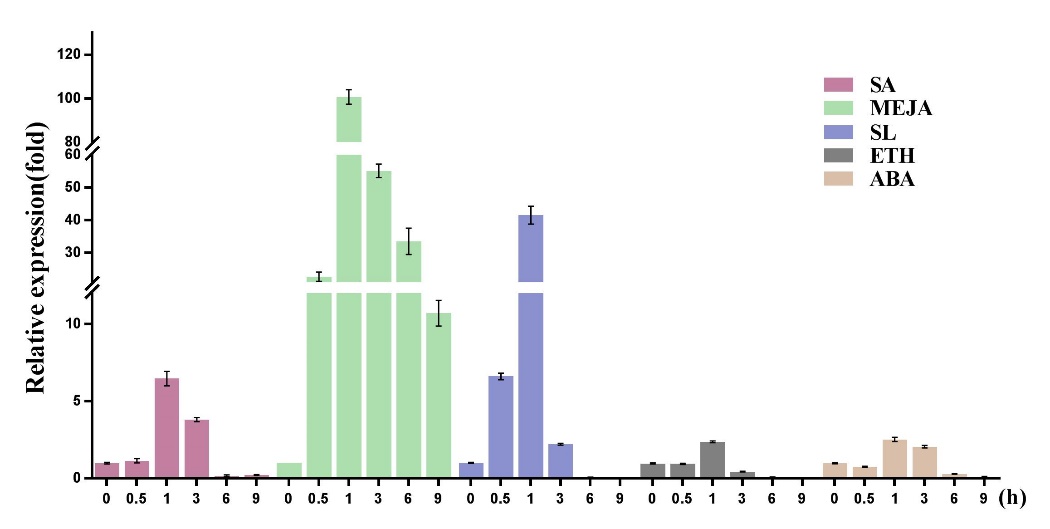


**Supplementary Fig. 15.** **Expression levels of *GauCBP1* in CSSL-1 seedlings treated with different hormones.** Tissues selected were true leaves of CSSL-1 seedlings (21-day) after sprayed with SA (5 mM), MEJA (100 μM ), SL (5 μM), ETH (100 μM ) and ABA (50 μM ) 0 h, 0.5 h, 1 h, 3 h, 6 h, 9 h.


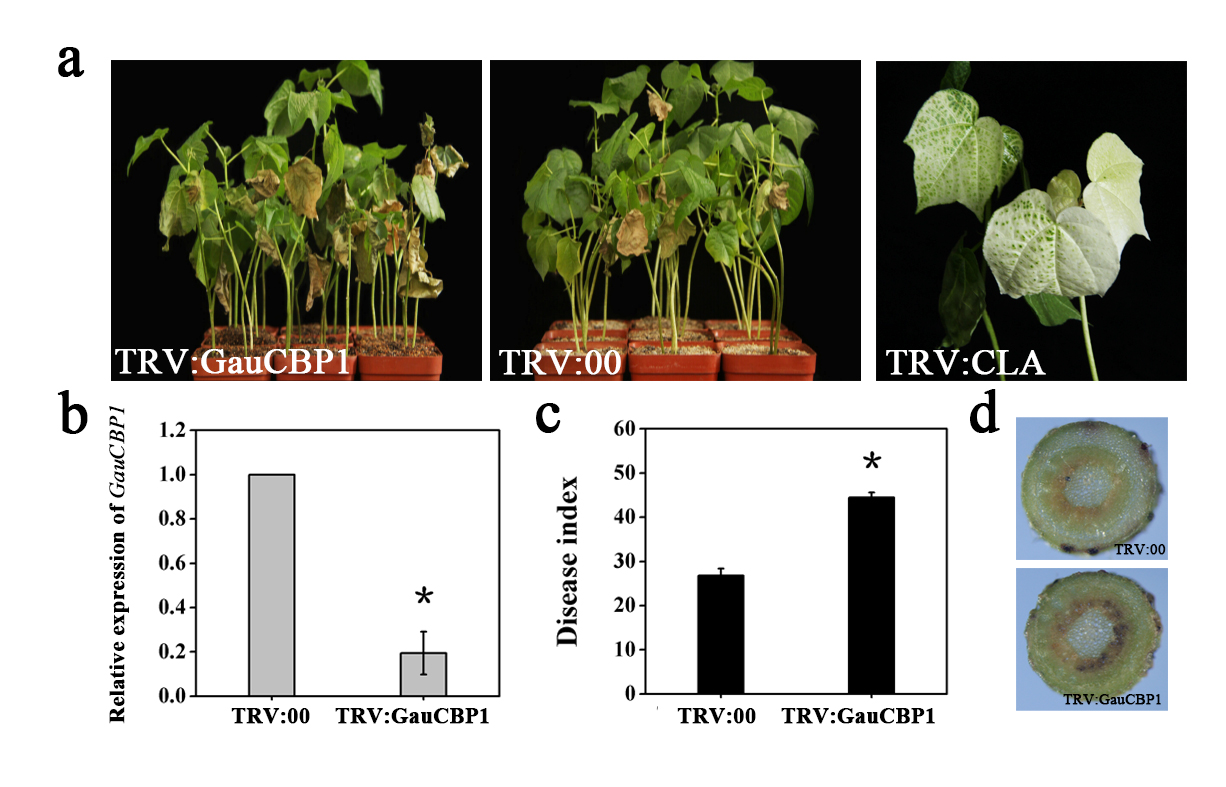


**Supplementary Fig. 16. The silencing of *GauCBP1* from *G. australe* compromised cotton resistance to *V. dahliae* in Xinhai 15. a,** Disease symptoms of TRV:GauCBP1(left), TRV:00 plants (center) under inoculation with *V. dahliae* strain V991 photographed at 17 dpi, and albino phenotype of the plants inoculated with TRV:CLA at 17 dpi (right). **b**, qRT-PCR analysis of the expression of *GauCBP1* in TRV:00 and TRV:GauCBP1. Statistical analyses were performed using Student’s t-test: *P< 0.05. **c**, The disease index and incidence rate in TRV:00 and TRV:GauCBP1 was measured at 17 dpi, respectively. Three biological replicates with at least 30 plants per replication. **d**, Section anatomy in stem was observed by *V. dahliae* treatment at 17 dpi in TRV:00 and TRV:GauCBP1. Bars, 1 mm.


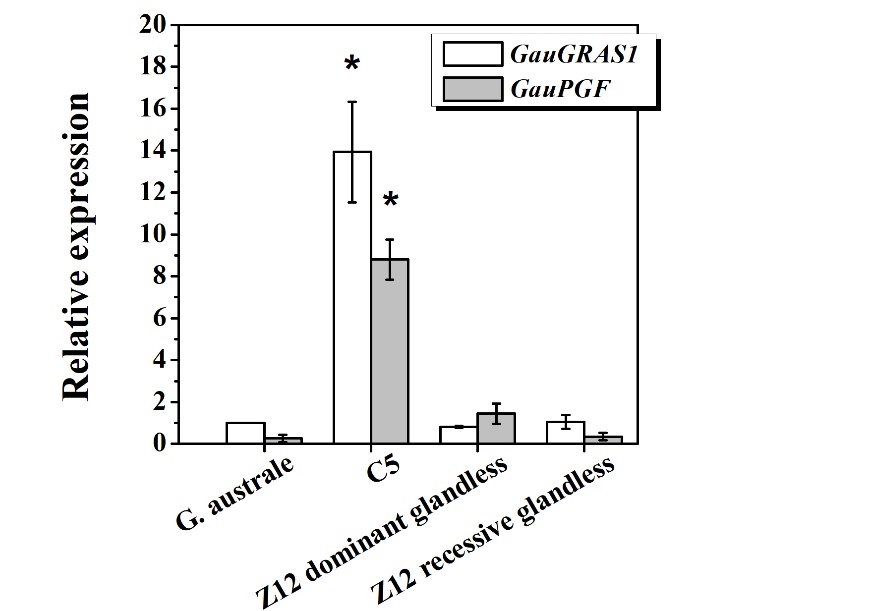


**Supplementary Fig. 17.** **Expression of *GauGRAS1 and GauPGF* in ovules of different gland materials.** Expression levels of *GRAS1* and *PGF* in ovules 10 dpa of *G. australe*, C5 (Jinxianduanguozhi, glanded *G.hirsutum*), Zhongmiansuo12 dominant glandless, Z Zhongmiansuo 12 recessive glandless. Error bars are s.d. of three biological repeats. *P<0.05; Student’s t-test, n = 3.


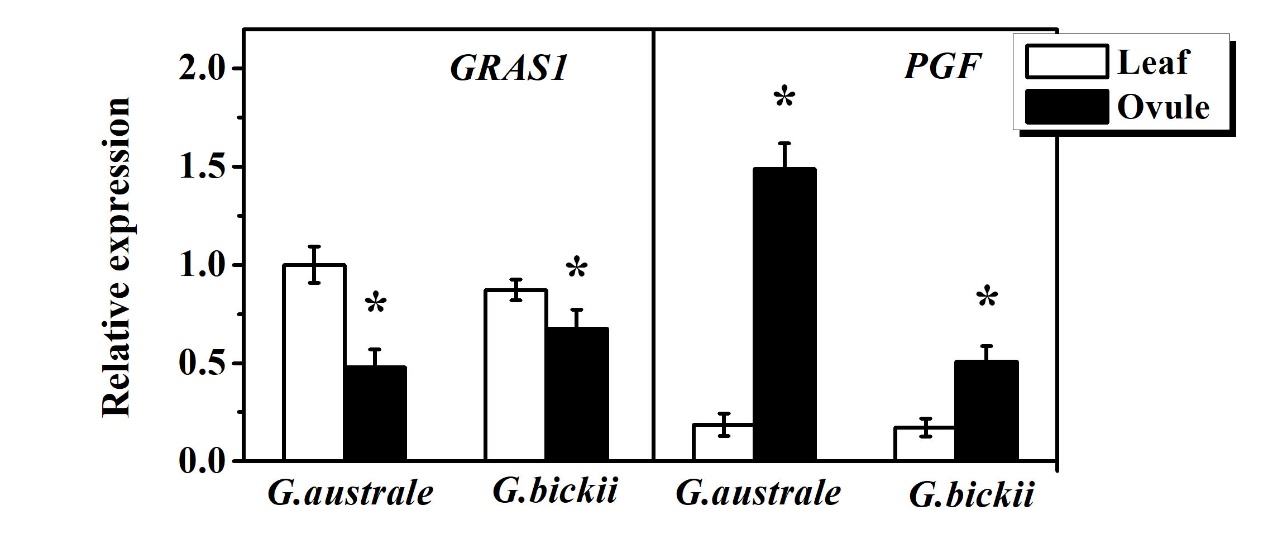


**Supplementary Fig. 18. Relative expression level of *GauPGF* and *GauGRAS1* gene in leaves (adult stage) and ovules (10 dpa) in two diploid G subgroup wild cotton species by qRT-PCR.**

**Supplementary Fig 19. Relative expression level of *GoPGF* and *GRAS1* gene in leaves and ovules in three diploid G subgroup wild cotton species, 16 dpa**


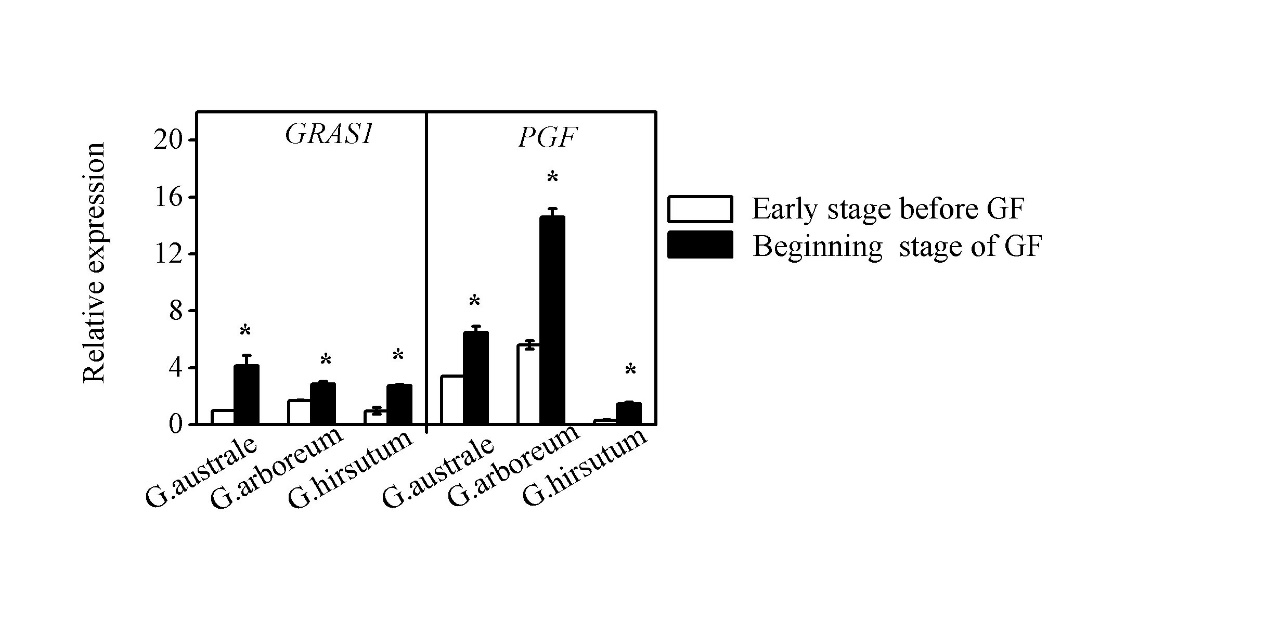


**Supplementary Fig.20. Relative expression level of *PGF* and *GRAS1* gene before and after GF (gland formation) during seed germination of three cotton species. Here the *G.hirsutum* is Xiangmian18 by RT-PCR.**


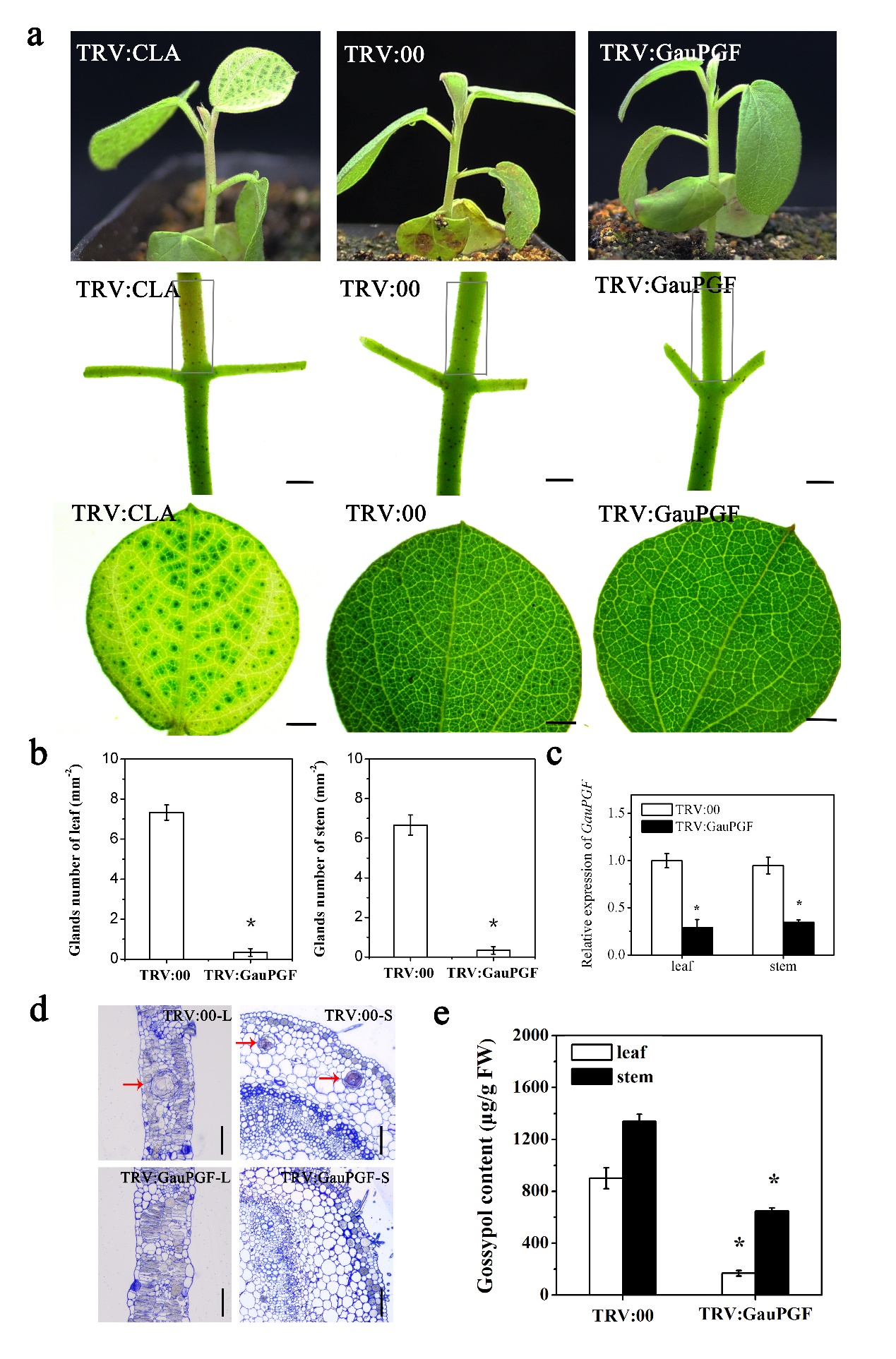


**Supplementary Fig. 21. Functional characterization of *GauPGF* by VIGS. (A) Phenotypes of *Gossypium australe* after *GauPGF* silencing by VIGS.** TRV:CLA and TRV:00 are the positive control and negative control. The gray box indicates glands in the stem, and the red arrow indicates glands on the leaf. Scale bars, 1 mm. (B) Statistical chart of the number of glands in leaves and stems. (C) The silencing efficiency of *GauPGF*. (D) Cavity observed in empty vector (TRV:00) leaves (TRV:00-L) and stems (TRV:00-S) but disappeared in the *GauPGF*-silenced plants (TRV:GauPGF-L and TRV:GauPGF-S). Scale bars, 100 μm. (E) Gossypol content in empty vector (TRV:00) and in the *GauPGF*-silenced leaves of *G. australe*, Error bars are s.d. of three biological repeats. *P<0.05; Student’s t-test, n = 3.


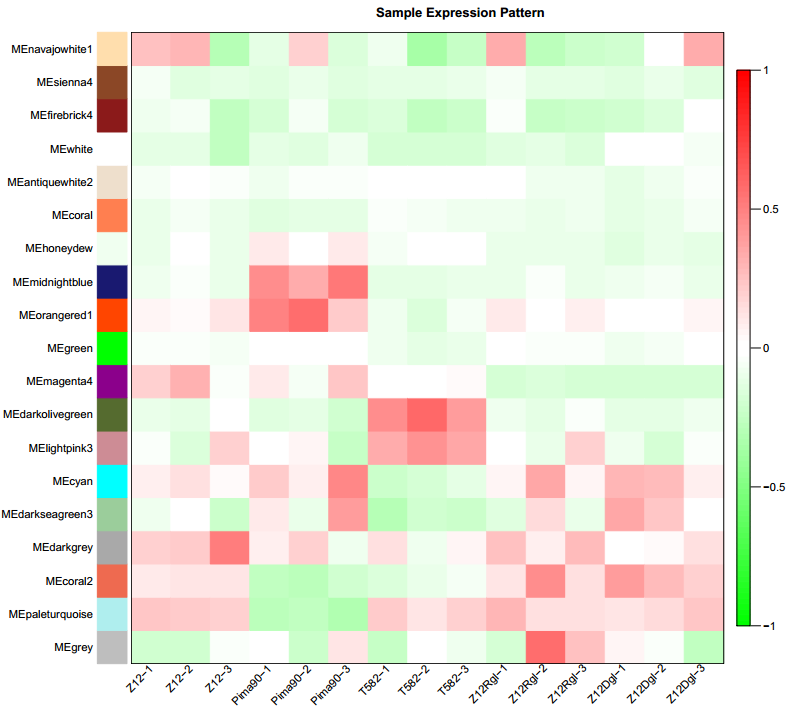


**Supplementary Fig. 22. Module-trait relations. Each row corresponds to a module eigengene, column to a transcriptome analysis of the embryos and leaf.** Glanded cotton varieties: Z12, Pima90, glandless cotton varieties: T582, Z12Dgl, Z12Rgl. MEmagenta4 was positively correlated with the presence/absence of gland.


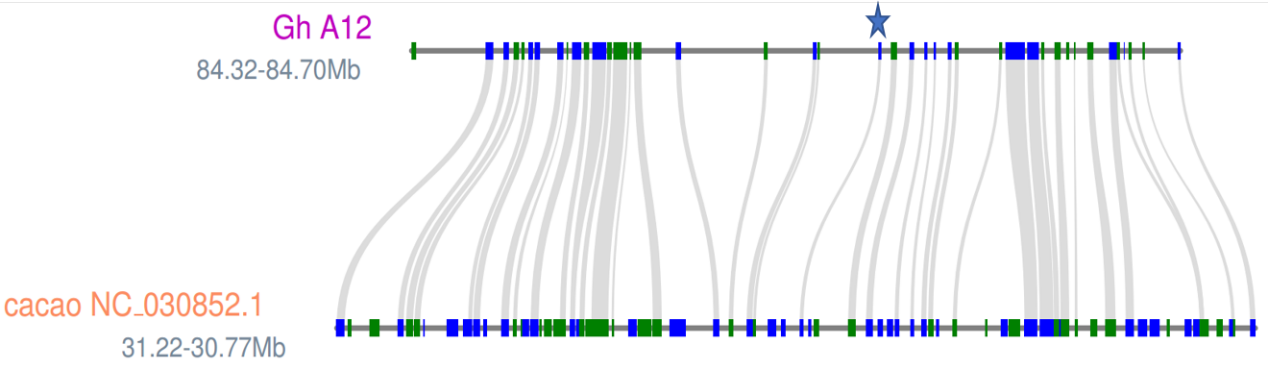


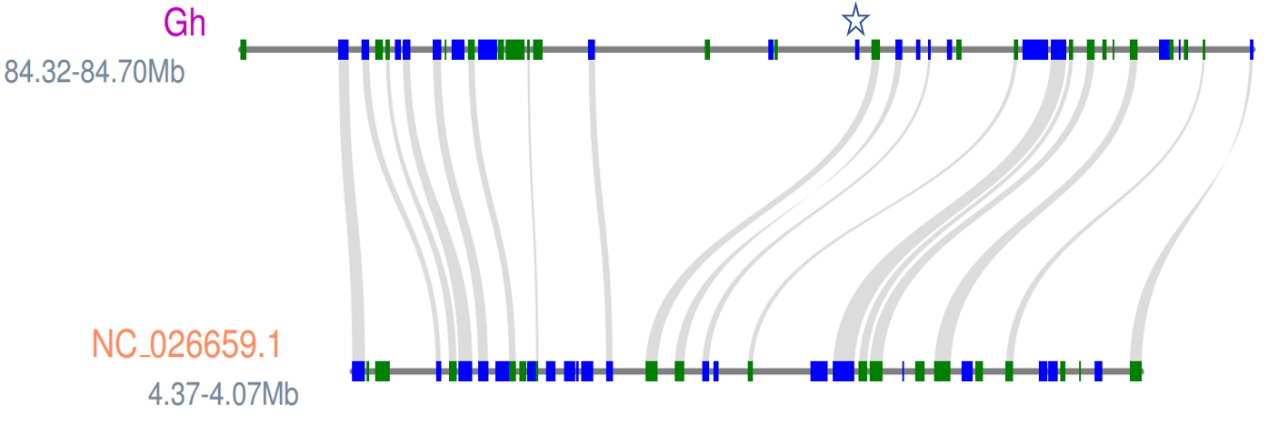


**Supplementary Fig. 23.** **Local collinearity based on forty genes adjacent to *GoPGF* of two example species**. The gene was supposed to be presence If both target gene and local collinearity exist. The star indicates GoPGF. Upper: local collinearity exists between *G. hirsutum* and *T. cacao*, lower: local collinearity does not exist between *G. hirsutum* and *Cucumis sativus*.


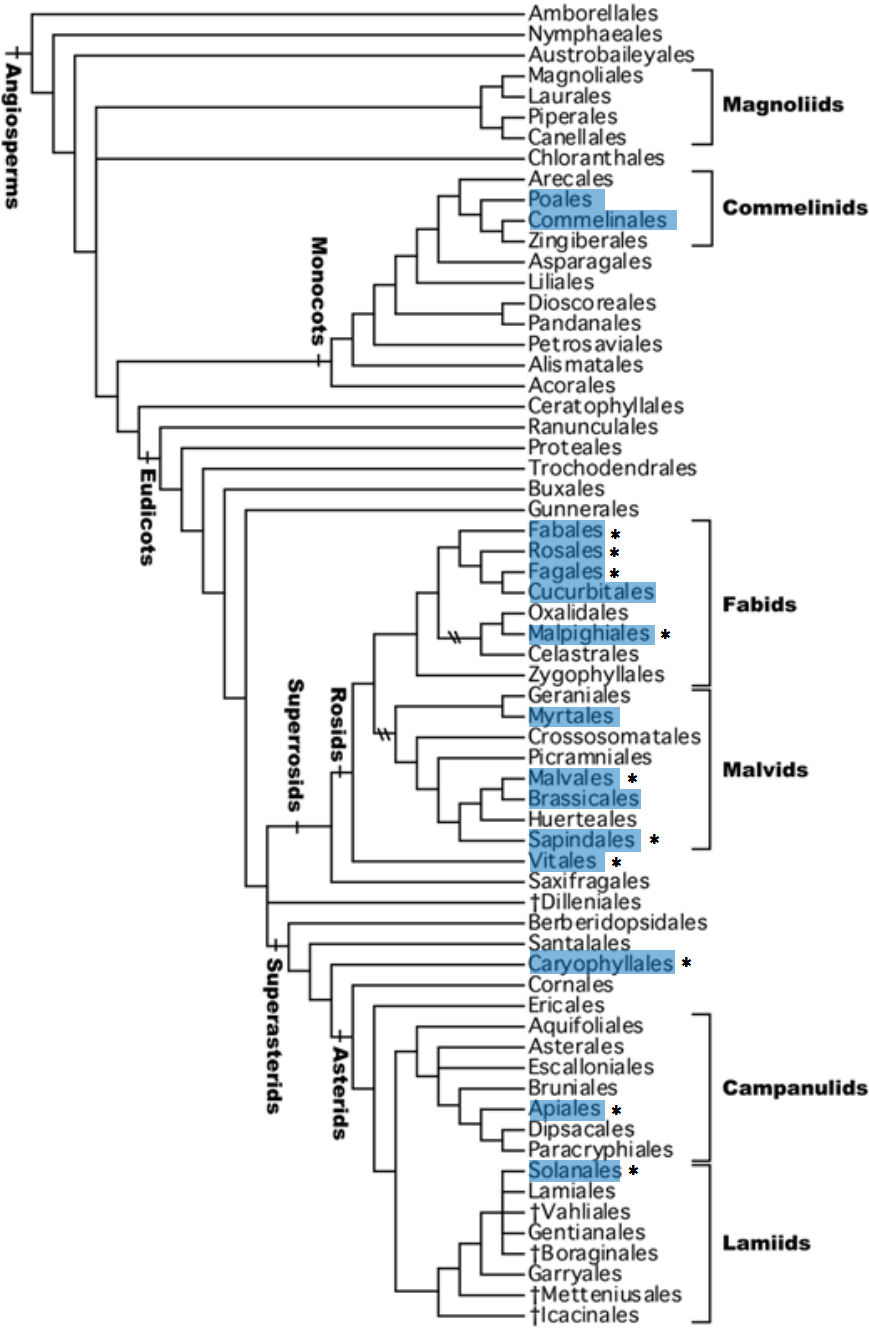


**Supplementary Fig. 24. Local collinearity of *GoPGF* between *G. hirsutum* and other genomes of angiosperm selected (blue) from the Angiosperm Phylogeny Group (APG) Ⅳ system**. Star indicates the presence of the existence of both target gene and local collinearity. Only blue with no star indicate the presence of local collinearity but absence of target gene. *GoPGF* gene does not exist in lower plants. There was collinearity in all the tested genomes of angiosperm selected from the Angiosperm Phylogeny Group (APG) Ⅳ system, while no *GoPGF* gene appeared in monocots. Collinearity existed in almost all the dicotyledonous plants no matter the glandular gland exists or not. A small number of dicotyledonous plants lost *GoPGF* in the entire family, such as the Brassicaceae, Cucurbitaceae.


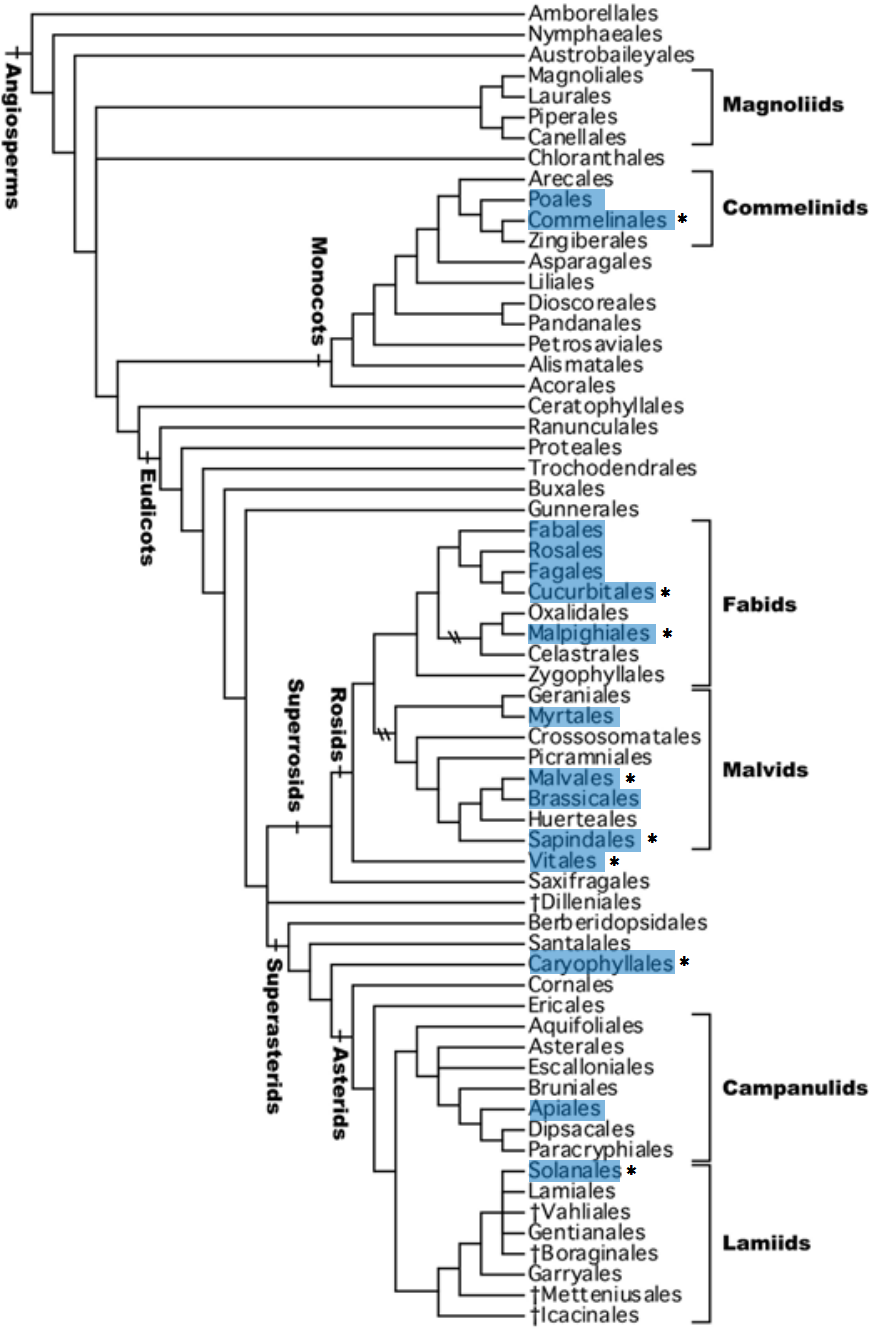


**Supplementary Fig. 25. Local collinearity of the *GRAS1* gene between *G. hirsutum* and other genomes of angiosperm selected (blue) from the Angiosperm Phylogeny Group (APG) Ⅳ system**. Star indicates the presence of the existence of both target gene and local collinearity. Only blue with no star indicate the presence of local collinearity but absence of target gene. The pattern of *GRAS1* gene is completely different from that of *GoPGF*, which exists even in monocotyledonous plants.
