# Supplementary material for: Genome sequencing of the Australian wild diploid species Gossypium australe highlights disease resistance and delayed gland morphogenesis
Source: Plant Biotechnol J. 2019 Sep 13;18(3):814–28. doi: 10.1111/pbi.13249 (PMC7004908; doi:10.1111/pbi.13249)
Supplement: Supplementary file 2 — Table S1 Genome assembly statistics for G. australe. Table S2 Assessment of sequence coverage of the G. australe genome assembly by homologous search using full‐length transcripts. Table S3 Number of genes with homology or functional classifications by different methods. Table S4 Analysis of non‐coding RNA genes in the G. australe genome. Table S5 Evaluation the quality of the annotation using the BUSCO method. Table S6 Summary and content analysis of different types of TEs in the G. australe genome. Table S7 Analysis of the content of major TE subfamilies in three Gossypium genomes. Table S8 Relative distribution (%) of Gypsy retrotransposon subgroups in the genomes of three Gossypium genomes. Table S9 Annotation of 31 genes that were up‐regulated profile genes in G. australe and down‐regulated profile genes in G. arboreum. Table S10 Annotation of 10 hub genes with top ten connectivity the magenta4 module of WGCNA analysis. Table S11 Annotation of genes adjacent to GoPGF co‐expressed in magenta4 module. Table S12 Primers used in VIGS and qRT‐PCR. [file PBI-18-814-s001.docx]

**Supplementary Table 1** Genome assembly statistics for *G. australe*

| **Assembly** | **Contig No.** | **Scaffold No.** | **Contig N50 (Mb)** | **Scaffold N50 (Mb)** | **Gap numbers** | **Gap size（Mb）** | **Assembly size (Gb)** |
| --- | --- | --- | --- | --- | --- | --- | --- |
| Pacbio | 1624 | - | 2.5 | - | - | - | 1.73 |
| Pacbio+Illumina | 1624 | 1097 | 2.5 | 3.59 | 527 | 0.78 | 1.73 |
| Optical Map | - | 246 | - | 138.25 | - | - | 1.85 |
| Pacbio+illumina+ Optical Map | 2485 | 691 | 1.82 | 145.15 | 1794 | 23.70 | 1.75 |
| Pacbio+illumina+ Optical Map+Hi-c | 2598 | 650 | 1.83 | 143.6 | 1948 | 23.65 | 1.75 |

**Supplementary Table 2** Assessment of sequence coverage of the *G. austral* genome assembly by homologous search using full-length transcripts

| **Sample** | **Total Hq_Isoform number** | **Total mapped (%)** | **Unmapped (%)** | **Multiple mapped (%)** | **Unique mapped (%)** |
| --- | --- | --- | --- | --- | --- |
| ovule | 75760 | 74430(98.24%) | 1330(1.76%) | 939(1.24%) | 73491(97.01%) |
| leaf | 82806 | 82475(99.60%) | 331(0.40%) | 1389(1.68%) | 81086(97.92%) |
| **Total** | **158566** | **156905(98.95%)** | **1661(1.05%)** | **2328(1.47%)** | **154577(97.48%)** |

**Supplementary Table 3** Number of genes with homology or functional classifications by different methods

|  | **Database** | **Number** | **Percent** |
| --- | --- | --- | --- |
| Total |  | 40855 | 100% |
| Annotated |  | 39666 | 97.09% |
|  | Nr | 39652 | 97.06% |
|  | Swissport | 27913 | 68.32% |
|  | KOG | 27263 | 66.73% |
|  | Kegg | 38512 | 94.27% |
| Unannotated |  | 1189 | 2.91% |

**Supplementary Table 4** Analysis of non-coding RNAs in the *G. australe* genome

| **Type** | | **Copy Number** | **Average Length(bp)** | **Total Length (bp)** | **Percentage of Genome (%)** |
| --- | --- | --- | --- | --- | --- |
| miRNA | | 399 | 126.67 | 50540 | 0 |
| tRNA | | 1292 | 74.17 | 95822 | 0.01 |
| rRNA | | | | | |
|  | 18s_rRNA | 86 | 1824.50 | 156907 | 0.01 |
|  | 28s_rRNA | 85 | 5933.91 | 504382 | 0.03 |
|  | 8s_rRNA | 1195 | 113.03 | 135069 | 0.01 |
| snRNA | | | | | |
|  | CD-box | 3189 | 106.24 | 338814 | 0.02 |
|  | HACA-box | 53 | 126.51 | 6705 | 0 |
|  | splicing | 146 | 146.08 | 21328 | 0 |
| **Total** | | **6445** |  | **1309567** | **0.08** |

**Supplementary Table 5** Evaluation the quality of the annotation using the BUSCO method

| **BUSCOs** | **Number** | **Percentage** |
| --- | --- | --- |
| Complete BUSCOs (C) | 1366 | 94.8% |
| Complete and single-copy BUSCOs (S) | 1181 | 82.0% |
| Complete and duplicated BUSCOs (D) | 185 | 12.8% |
| Fragmented BUSCOs (F) | 16 | 1.1% |
| Missing BUSCOs (M) | 58 | 4.1% |
| Total BUSCO groups searched | 1440 | 100% |

**Supplementary Table 6** Summary and content analysis of different types of TEs in the *G. australe* genome

|  | **Repbase TEs** | |  | **TE protiens** | | |  | **De novo** | |  | **Combined TEs** | |  |
| --- | --- | --- | --- | --- | --- | --- | --- | --- | --- | --- | --- | --- | --- |
| **Type** | **Length (Bp)** | **% in genome** | | **Length (Bp)** | | **% in genome** |  | **Length (Bp)** | **% in genome** | | **Length (Bp)** | **% in genome** |  |
| DNA | 21204370 | 1.225064 | | 14742868 | 0.851756 | | | 19290457 | 1.114489 | | 34880816 | 2.015209 | |
| LINE | 2504923 | 0.144720 | | 17332967 | 1.001397 | | | 5530577 | 0.319524 | | 24033282 | 1.388502 | |
| SINE | 9262 | 0.000535 | | 0 | 0 | | | 0 | 0 | | 9262 | 0.000535 | |
| LTR | 224825533 | 12.98910 | | 343997165 | 19.874140 | | | 1084937012 | 62.681290 | | 1181575281 | 68.264482 | |
| Other | 17229 | 0.000995 | | 0 | 0 | | | 0 | 0 | | 17229 | 0.000995 | |
| Unknown | 62413 | 0.003606 | | 0 | 0 | | | 69959866 | 4.041870 | | 70019891 | 4.045338 | |
| **Total** | **247256807** | **14.285050** | | **375266139** | **21.680670** | | | **1165972788** | **67.363060** | | **1272213756** | **73.501041** | |

**Supplementary Table 7** Analysis of the content of major TE subfamilies in three *Gossypium* genomes

|  | ***G. autrale*** | ***G. arboreum*** | ***G. raimondii*** |
| --- | --- | --- | --- |
| LTR/Copia | 4.9 | 5.5 | 11.1 |
| LTR/Gypsy | 56.5 | 55.8 | 33.8 |
| LINE | 1.4 | 1.2 | 1.5 |
| SINE | <0.01 | 0.01 | <0.01 |
| DNA/hAT | 0.2 | 0.2 | 0.6 |
| DNA/TcMar | 0.02 | <0.01 | <0.01 |
| DNA/Helitron | 0.03 | <0.01 | <0.01 |
| DNA/MULE | 1.5 | 0.4 | 1.2 |
| DNA/PIF | 0.2 | 0.03 | 0.3 |
| Other | 6.4 | 4.5 | 8.5 |

**Supplementary Table 8** Relative distribution (%) of gypsy retrotransposon subgroups in the genomes of three *Gossypium* genomes

|  | ***G. autrale*** | ***G. arboreum*** | ***G. raimondii*** |
| --- | --- | --- | --- |
| Tat | 2.78% | 0.82% | 9.74% |
| Athila | 3.96% | 1.73% | 27.27% |
| CRM | 88.35% | 92.24% | 16.88% |
| Reina | 0.18% | 0.82% | 13.64% |
| Del | 0.12% | 0.18% | 4.55% |
| Galadriel | 4.61% | 4.20% | 27.92% |

**Supplementary Table 9** Annotation of 31 genes that were up-regulated or down-regulated profile genes in *G. arboreum*

| **Gene ID** | **Function annotation** |
| --- | --- |
| GAUG00000086 | Tropinone reductase homolog At1g07440-like |
| GAUG00004824 | Uncharacterized protein |
| GAUG00007269 | Transcription factor bHLH19-like isoform |
| GAUG00007489 | L-ascorbate oxidase homolog protein |
| GAUG00007771 | Proline dehydrogenase 2 |
| GAUG00016184 | ABC transporter B family member 4-like protein |
| GAUG00017592 | Cysteine-rich repeat secretory protein 38-like protein |
| GAUG00020453 | Transient receptor potential cation channel subfamily A member 1-like Protein |
| GAUG00020868 | BURP domain-containing protein 3-like |
| GAUG00020869 | organ-specific protein P4-like |
| GAUG00021443 | (R,S)-reticuline 7-O-methyltransferase-like |
| GAUG00023497 | Uncharacterized protein |
| GAUG00024767 | Suppressor of disruption of TFIIS-like |
| GAUG00025390 | Lysine histidine transporter-like 8 |
| GAUG00025508 | Glycine-rich protein 2-like |
| GAUG00025864 | LRR receptor-like serine/threonine-protein kinase |
| GAUG00028019 | Calmodulin-binding family protein (CamBP2) mRNA, complete cds |
| GAUG00029304 | 17.6 kDa class II heat shock protein-like |
| GAUG00029404 | pathogenesis-related protein STH-2-like |
| GAUG00030074 | Pathogenesis-related protein STH-2-like |
| GAUG00031473 | ECERIFERUM 1-like |
| GAUG00033115 | Pyruvate kinase 1 |
| GAUG00033840 | Putative receptor-like protein kinase |
| GAUG00034976 | Probable leucine-rich repeat receptor-like protein kinase |
| GAUG00037396 | Protein NRT1/ PTR FAMILY 2.7-like |
| GAUG00038053 | Receptor-like protein kinase FERONIA |
| GAUG00040000 | Alpha carbonic anhydrase 1 |
| GAUG00044606 | Uncharacterized protein |
| GAUG00046021 | Rust resistance kinase Lr10-like |
| GAUG00046774 | Homeobox protein knotted-1-like 2 |
| GAUG00050618 | 1-aminocyclopropane-1-carboxylate oxidase homolog 1-like |

**Supplementary Table 10** Annotation of 10 hub genes with top ten connectivity the magenta4 module of WGCNA analysis

| **Gene ID** | **Function annotation** |
| --- | --- |
| Gohir.D03G138300 | lactoylglutathione lyase family protein / glyoxalase I family protein |
| Gohir.A13G205300 | 2-oxoglutarate (2OG) and Fe(II)-dependent oxygenase superfamily protein |
| Gohir.A05G249300 | laccase 14 |
| Gohir.D13G210300 | 2-oxoglutarate (2OG) and Fe(II)-dependent oxygenase superfamily protein |
| Gohir.D05G310700 | cytochrome P450, family 82, subfamily C, polypeptide 4 |
| Gohir.A04G046800 | cytochrome P450, family 71, subfamily B, polypeptide 34 |
| Gohir.A03G012200 | cytochrome P450, family 706, subfamily A, polypeptide 6 |
| Gohir.D05G201200 | cytochrome P450, family 82, subfamily C, polypeptide 4 |
| Gohir.A07G074300 | glutathione S-transferase phi 12 |
| Gohir.D12G159900 | Transmembrane protein 97, predicted |

**Supplementary Table 11** Annotation of genes adjacent to GoPGF co-expressed in magenta4 module

| **Gene ID** | **Function annotation** |
| --- | --- |
| Gohir.D12G159900 | Transmembrane protein 97, predicted |
| Gohir.D12G159600 | Transmembrane protein 97, predicted |
| Gohir.D12G198900 | Integrase-type DNA-binding superfamily protein |
| Gohir.D12G264900 | cytochrome P450, family 706, subfamily A, polypeptide 4 |
| Gohir.D12G241700 | Basic helix-loop-helix (bHLH) DNA-binding family protein |
| Gohir.D12G207800 | GHMP kinase family protein |
| Gohir.D12G227000 | hydroxymethylglutaryl-CoA synthase / HMG-CoA synthase / 3-hydroxy-3-methylglutaryl coenzyme A synthase |
| Gohir.D12G148600 | ATP-citrate lyase A-1 |

**Supplementary Table 12** Primers used in VIGS and qRT-PCR

| **Primer** | **Sequence (5’-3’)** |
| --- | --- |
| V-*GauCCD7*-F | AGAAGGCCTCCATGGGGATCCACGTCTGAGCCGATCACACTCC |
| V-*GauCCD7*-R | GAGACGCGTGAGCTCGGTACCTCGCCACGAGCCGGTCAC |
| V-*GauCBP1*-F | CCGGAATTCGTTGGAGATGGGAAAGAAGT |
| V-*GauCBP1*-R | CGGGGTACCCTTCCGTTGGATGATAATGAC |
| V-*GauGRAS1*-F | CGCGGATCCCGGCAGATCCCGAGTGAAATAG |
| V-*GauGRAS1*-R | CGGGGTACCCCAGAATCAGGAAAACCACCCA |
| V-*GauPGF*-F | AGAAGGCCTCCATGGGGATCCTCATCATGGAAGACTGGGGC |
| V-*GauPGF*-R | GAGACGCGTGAGCTCGGTACCTTTTCGACCGGCCGCTGG |
| q-*GauCBP1*-F | GATTGGTTGCTCGTGATGG |
| q-*GauCBP1*-R | GCTTGCCCGTATGAGTTGT |
| q-*GauCCD7*-F | TTTATGGGAAGGTGGTG |
| q-*GauCCD7*-R | GTTTCTCGGCGGATGG |
| q-*GauGRAS1*-F | ACGCAGATGACCATTCCT |
| q-*GauGRAS1*-R | CCCAACAGTAGCACCAAG |
| q-*GauPGF*-F | CACCCTTCAACTCACTAA |
| q-*GauPGF*-R | GCAGAGGCGAAATCCC |
| q-*UBQ7*-F | GGTTTCCGAATGGTCTTTTG |
| q-*UBQ7*-R | TTTAGCCACTGCATCACAGC |
